# A frameshift mutation in MOCOS is associated with familial renal syndrome (xanthinuria) in Tyrolean Grey cattle

**DOI:** 10.1186/s12917-016-0904-4

**Published:** 2016-12-05

**Authors:** Leonardo Murgiano, Vidhya Jagannathan, Christian Piffer, Inmaculada Diez-Prieto, Marilena Bolcato, Arcangelo Gentile, Cord Drögemüller

**Affiliations:** 1 Institute of Genetics, Vetsuisse Faculty, University of Bern, Bern, Switzerland; 2 Servizio Veterinario dell’Azienda Sanitaria dell’Alto Adige, Bozen, Italy; 3 Laboratory on Urolithiasis Research, Department of Medicine, Surgery and Anatomy, Universidad de León, León, Spain; 4 Department of Veterinary Medical Sciences, University of Bologna, Ozzano dell’Emilia, Italy

**Keywords:** Bovine, Congenital disease, Hereditary, Kidney, Rearing success

## Abstract

**Background:**

Renal syndromes are occasionally reported in domestic animals. Two identical twin Tyrolean Grey calves exhibited weight loss, skeletal abnormalities and delayed development associated with kidney abnormalities and formation of uroliths. These signs resembled inherited renal tubular dysplasia found in Japanese Black cattle which is associated with mutations in the *claudin 16* gene. Despite demonstrating striking phenotypic similarities, no obvious presence of pathogenic variants of this candidate gene were found. Therefore further analysis was required to decipher the genetic etiology of the condition.

**Results:**

The family history of the cases suggested the possibility of an autosomal recessive inheritance. Homozygosity mapping combined with sequencing of the whole genome of one case detected two associated non-synonymous private coding variants: A homozygous missense variant in the uncharacterized *KIAA2026* gene (g.39038055C > G; c.926C > G), located in a 15 Mb sized region of homozygosity on BTA 8; and a homozygous 1 bp deletion in the *molybdenum cofactor sulfurase* (*MOCOS*) gene (g.21222030delC; c.1881delG and c.1782delG), located in an 11 Mb region of homozygosity on BTA 24. Pathogenic variants in *MOCOS* have previously been associated with inherited metabolic syndromes and xanthinuria in different species including Japanese Black cattle. Genotyping of two additional clinically suspicious cases confirmed the association with the *MOCOS* variant, as both animals had a homozygous mutant genotype and did not show the variant *KIAA2026* allele. The identified genomic deletion is predicted to be highly disruptive, creating a frameshift and premature termination of translation, resulting in severely truncated MOCOS proteins that lack two functionally essential domains. The variant *MOCOS* allele was absent from cattle of other breeds and approximately 4% carriers were detected among more than 1200 genotyped Tyrolean Grey cattle. Biochemical urolith analysis of one case revealed the presence of approximately 95% xanthine.

**Conclusions:**

The identified *MOCOS* loss of function variant is highly likely to cause the renal syndrome in the affected animals. The results suggest that the phenotypic features of the renal syndrome were related to an early onset form of xanthinuria, which is highly likely to lead to the progressive defects. The identification of the candidate causative mutation thus enables selection against this pathogenic variant in Tyrolean Grey cattle.

**Electronic supplementary material:**

The online version of this article (doi:10.1186/s12917-016-0904-4) contains supplementary material, which is available to authorized users.

## Background

Sporadic cases of inherited renal syndromes have been reported in domestic animals including cattle. In Danish Holsteins and Danish Red dairy cattle, an autosomal recessive form of renal lipofuscinosis has been described, accompanied by renal dysfunction and reduced longevity (OMIA 001407–9913) [1]. A single case of renal amyloidosis has been described in Iranian cattle (OMIA 000040–9913) [2] and various forms of renal dysplasia have been reported in other cattle (OMIA 001135–9913) [3–9]. In Japanese Black cattle, renal dysplasia occurred due to two independent autosomal recessive mutations of the *claudin 16* gene (*CLDN16*), but no phenotypic differences between both types were reported [10–12]. Members of the claudin gene family play important roles in the formation of tight junctions in the kidney [13]. In Japanese Black cattle, inherited xanthinuria is associated with growth retardation and death at approximately 6 months of age (OMIA 001819–9913) [14]. This autosomal recessive inherited disease has been associated with a 3 bp deletion in the coding region of the *molybdenum cofactor sulfurase* (*MOCOS*) gene*.* In a single genetically uncharacterized Galician Blond beef calf, signs of listlessness and weight loss and renal failure with bilateral nephrolithiasis, composed of 100% xanthine were reported [15].

In a previous report [16] the authors described kidney abnormalities in two eight months old female Alpine Grey (better known as Tyrolean Grey) twin cattle presenting a number of signs: growth retardation, overgrowth of hooves, gradual loss of weight and impaired skeletal development, despite a normal or only slightly decreased appetite and intense vitamin integration.

Clinical biochemistry data e.g. associated with variable blood phosphate concentrations indicated a renal failure. Pathological examination of the animals showed hypotrophic, firm and pale kidneys, with a roughened and granular surface. Subsequent histology showed interstitial infiltrates of immature mesenchymal tissue and disseminated mineralization. Mild dilation of the pelvis with free yellow calculi and disseminated medullar mineralization were also observed. Small stones (0.3 to 0.5 cm in diameter) were also found in the ureters and in the bladder. The observed renal syndrome in Tyrolean Grey cattle resembled inherited renal tubular dysplasia in Japanese Black cattle. Despite phenotypic similarities, no mutation in *CLDN16* was identified [16]. Therefore, the aim of this study was to use whole genome sequencing to unravel the genetic etiology of this condition.

## Methods

### Animals and SNP genotyping

Initially, blood samples were collected from two affected twin calves and their parents. Genotyping of these four animals was performed using the BovineHD BeadChip (Illumina), including 777,962 evenly distributed SNPs, at GeneSeek. PLINK software [17] was used to search for extended intervals of homozygosity with shared alleles as described previously [18]. Additionally, archived DNA samples from 1201 normal Tyrolean Grey cattle were used for genotyping the *MOCOS* variant. During the course of the review process of this paper, 3 additional blood samples were taken from two clinically suspicious cases and their dam. For the mutation analysis and comparison of sequencing data, 106 normal cattle from 20 genetically diverse *Bos taurus* breeds were used: Angler (*n* = 5), Angus (*n* = 3), Brown Swiss (*n* = 8), Charolais (*n* = 1), Chianina (*n* = 1), Cika (*n* = 1), Danish Red Dairy (*n* = 3), Eringer (*n* = 2), Galloway (*n* = 2), Hereford (*n* = 1), Hinterwalder (*n* = 3), Holstein (*n* = 47), Limousin (*n* = 4), Pezzata Rossa Italiana (*n* = 1), Piemontese (*n* = 1), Romagnola (*n* = 5), Scottish Highland (*n* = 2), Simmental (*n* = 12), Tyrolean Grey (*n* = 1), and Vorderwalder (*n* = 3).

### Whole genome re-sequencing

A fragment library with a 260 bp insert size was prepared and one lane of Illumina HiSeq2500 paired-end reads collected (2 × 100 bp); the fastq files were created using Casava 1.8 (illumina). Sequence reads were then mapped to the reference cow genome assembly UMD3.1 as previously described [19], at average sequence coverage of 19-fold. The SAM file generation, conversion to BAM, duplicate detection, variant calling (data for each sample was obtained in variant call format, version 4.0), and variant effect prediction were carried out as described previously [20]. The genome data has been made freely available under accession PRJEB11962 at the European Nucleotide Archive [21].

### Genotyping

The *MOCOS* variant was genotyped by Sanger sequencing of a 287 bp PCR product using a forward primer (5-CATCATTTCACTTCCTTTTGGA-3) and a reverse primer (5-TAGGTGATCAGGTGGCCTCT-3) flanking the *MOCOS* variant. PCR products were amplified with AmpliTaqGold360Mastermix (Life Technologies) and the products directly sequenced using the PCR primers on an ABI 3730 capillary sequencer (Life Technologies). Sequence data were analyzed using Sequencher 5.1 (GeneCodes). In addition, fragment size analyses were performed for the genotyping of the *MOCOS* variant on the ABI 3730 and analyzed with the GeneMapper 4.0 software (Life Technologies).

### Urolith analysis

The uroliths were washed with deionized distilled water and dried in an oven at 45 °C for 48 h. After visual inspection of the external surface by stereomicroscope, appearance, color and size were recorded, stones weighed and one of the largest stones cut (to check for layers that were analyzed independently). The test samples were ground in an agate pestle and mortar to obtain a homogenous powder. A small amount of the powder (approximately 2 mg) was mixed with 200 mg of potassium bromide and this mixture pressed in a ten-tone press to form a thin pill/pellet. Fourier transform infrared (FT-IR) spectroscopy of this pellet was performed in a spectrophotometer (FT-IR 2000, Perkin Elmer, United Kingdom) and the obtained spectrum compared with a specific library for uroliths (IR Kidney Stones 1668 Spectra, Nikodom, Czech Republic).

## Results and discussion

### Pedigree analysis and homozygosity mapping suggests a recessive inheritance

**Fig. 1:**
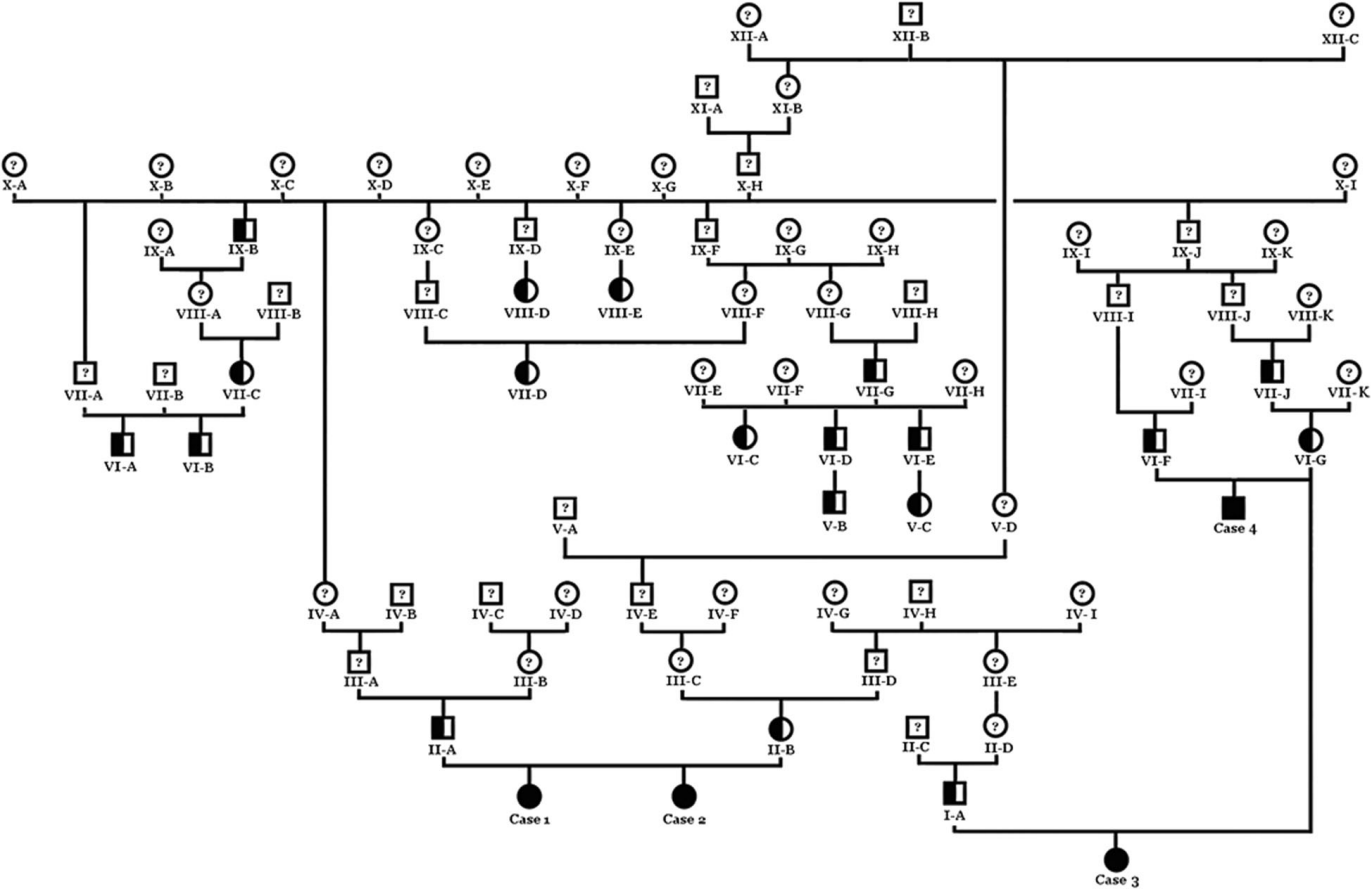
Family tree of the affected Tyrolean Grey cattle with renal syndrome (xanthinuria). Males are represented by *squares*, females by *circles*. Affected animals are shown with fully *black symbols. Half-filled symbols* represent healthy obligate heterozygous carriers and *open symbols with a question mark* represent relatives with an unknown genotype. Cases 1 and 2 are the initially studied twin cases, whereas case 3 and 4 are the additional cases reported later

**Fig. 2:**
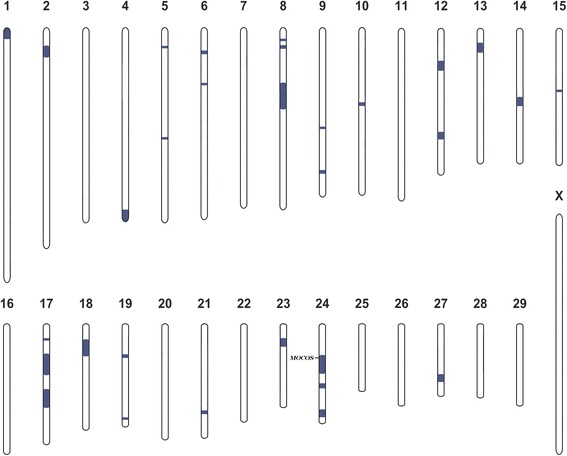
Homozygosity mapping in Tyrolean Grey cattle with renal syndrome (xanthinuria). Extended segments of private homozygosity in both cases are shown in *blue*. Note, that the *MOCOS* gene is located in an 11 Mb sized homozygous regions on chromosome 24

The pedigree of the established Tyrolean Grey cattle family was consistent with a possible monogenic autosomal recessive inheritance (Fig. 1). Both parents were healthy and could be traced back to a single common male ancestor born ~40 years ago. The genotypes of more than 770,000 evenly spaced SNPs showed that the affected twins were monozygotic twins with an identity by state (IBS) of 100%. Since we assumed a homozygous recessive condition, the affected calves were expected to be identical by descent (IBD) and homozygous for the causative mutation and flanking chromosomal segments. The search for extended regions of homozygosity with simultaneous allele sharing was carried out, and the results compared with the homozygous region between the cases and the parents. Thereby, 29 genome regions greater than 1 Mb were identified where both affected animals were homozygous, but the parents were not (Fig. 2, Additional file 1).

Although it was assumed that recessive inheritance was considered most likely, a second possibility that the renal syndrome was due to a spontaneous dominant acting mutation could not be excluded. In fact, since after genotyping the twins were identified as identical twins, the causative mutation could have occurred *de novo* in the parental germinal cells. This alternative hypothesis was acknowledged in further analyses.

### Whole genome sequencing reveals a deleterious *MOCOS* mutation

Due to the severe effect of the mutation, we suggested that most likely a non-synonymous loss of function mutation affecting the coding sequence of a gene would be responsible for the renal syndrome. The whole genome of case 1 was sequenced, and initially the variant calls were filtered according to the most likely scenario of recessive inheritance. A total of 36,940 homozygous SNPs and short indel variants lying in the coding regions of annotated genes were called across the entire genome. A triple-step filtering to search for private variants was applied: (*I*) Only homozygous variants falling into the homozygous IBD regions presented above were considered; (*II*) A comparison was then made between the remaining variants and variant data of 106 cow genomes of various different cattle breeds that were sequenced in our laboratory in the course of other ongoing studies; (*III*) Lastly, the remaining variants were compared with the run4 variant database of the 1000 bull genome project [22] including 1119 additional genomes of different breeds. Finally, the number of private homozygous variants was dropped to 435. These included two non-synonymous variants (Additional file 2): A missense variant in exon 8 of the *KIAA2026* gene (c.926C > G; p.S309C), located in a 15 Mb sized region of homozygosity on BTA 8 (g.39038055C > G); and a 1 bp deletion in exon 9 of the *molybdenum cofactor sulfurase* (*MOCOS*) gene, located in an 11 Mb region of homozygosity on BTA 24 (g.21222030delC). The function of KIIAA2026 is not well characterized, however recent studies identified KIIAA2026 as interacting with ubiquitin-like UBTD1 in a yeast-2-hybrid screen [23], and a possible role in cancer [24]. On the other hand, the MOCOS enzyme is known to affect kidney metabolism and kidney development and a *MOCOS* mutation was previously shown to be associated with a renal condition in cattle, specifically xanthinuria, characterized by developmental problems, renal failure and the presence of kidney stones [14]. Therefore *MOCOS* represents a very good candidate gene for the condition, and the detected *MOCOS* variant is much more likely to be responsible for the observed phenotype.

**Fig. 3:**
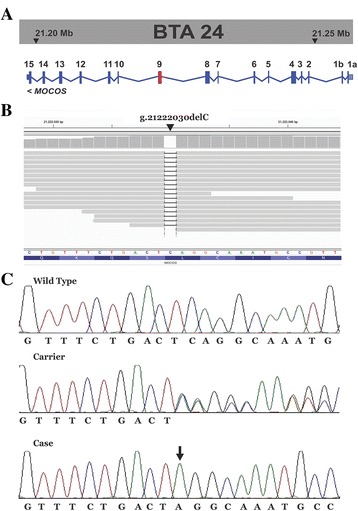
A 1 bp deletion in exon 9 of *MOCOS* associated with renal syndrome (xanthinuria) in Tyrolean Grey cattle. **a** *MOCOS* gene and its position on bovine chromosome24. Note the two alternative first exons (1a and 1b), leading to the alternate transcripts (*MOCOS-201* and *MOCOS-202*, shown in Fig. 6). **b** Screenshot of the next generation sequencing reads mapped against the reference sequence. Note the C deletion in exon 9. **c** Electropherograms of a control Tyrolean Grey cattle (wild type), a parent of one of the affected twins (heterozygous carrier), and one affected case (homozygous). The arrow indicates the position of the 1 bp deletion

The *MOCOS* variant was confirmed by Sanger sequencing (Fig. 3). The 1 bp deletion in the bovine *MOCOS* gene affects both annotated transcripts [25]: ENSBTAT00000048768 (*MOCOS-201*; c.1881delG) and ENSBTAT00000065375 (*MOCOS-202*; c.1782delG). For both *MOCOS* transcripts, the deletion is predicted to cause a frameshift introducing premature stop codons (p.Ser628Valfs9* and p.Ser595Valfs9*).

The possibility that the condition was caused by a recent *de novo* mutation could not be excluded, therefore the private heterozygous variants present in the genome of the sequenced case were also analyzed. The number of heterozygous variants identified in the coding regions of the affected case were 124,393; these were then filtered against the in house 106 control genomes. Of the remaining 445 variants, 328 were subsequently excluded because of their presence in the 1000 bull genome variant database. Finally, a total of 59 private heterozygous coding variants remained. These did not contain any non-synonymous variants and no other variants in genes which might represent suitable candidates for the renal phenotype (Additional file 3).

Thus, the final conclusion was that among all the possible variants, the recessive inherited deleterious *MOCOS* variant represented the most probable causative mutation for the renal syndrome. Therefore, the observed renal syndrome most likely represents a xanthinuria which causes the severe (early-onset) metabolical signs and the occurrence of kidney stones. Genotyping this variant in an extended cohort of Tyrolean Grey cattle confirmed that the *MOCOS* deletion was associated perfectly with the condition: only the two twins carried two copies of the mutation, both parents were heterozygous carriers and none of the 1201 controls showed the homozygous mutant genotype (Table 1). A total of 50 genotyped controls (~4%) were heterozygous carriers. In each case of 13 carriers with available pedigree records a relationship to the previously identified male ancestor was noticed (Fig. 1).

**Table 1:** Association of the 1 bp deletion in *MOCOS* with the renal syndrome (xanthinuria) in Tyrolean Grey cattle

	*MOCOS*		
	*c.1881delG /c.1782delG*		
	G/G	G/del	del/del
Affected animals			4
Obligate carriers		3	
Tyrolean Grey population controls	1151	50	
Controls of other breeds^a^	1225		
Total	2376	53	4

^a^106 in-house controls and 1119 genomes of the 1000 bull genome project

### Additional cases confirmed *MOCOS-*association and kidney stone analysis supporting xanthinuria diagnosis

**Fig. 4:**
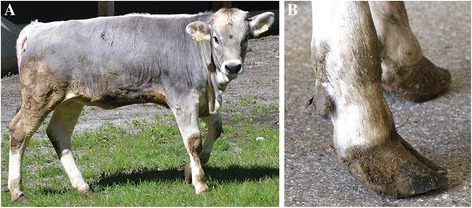
Six-month-old Tyrolean Grey heifer with renal syndrome (xanthinuria). **a** The affected animal showed retarded growth and discontinuous disturbed appetite. **b** Note the overgrown hooves. The owner reported an exaggerated growth and therefore cut the hooves

**Fig. 5:**
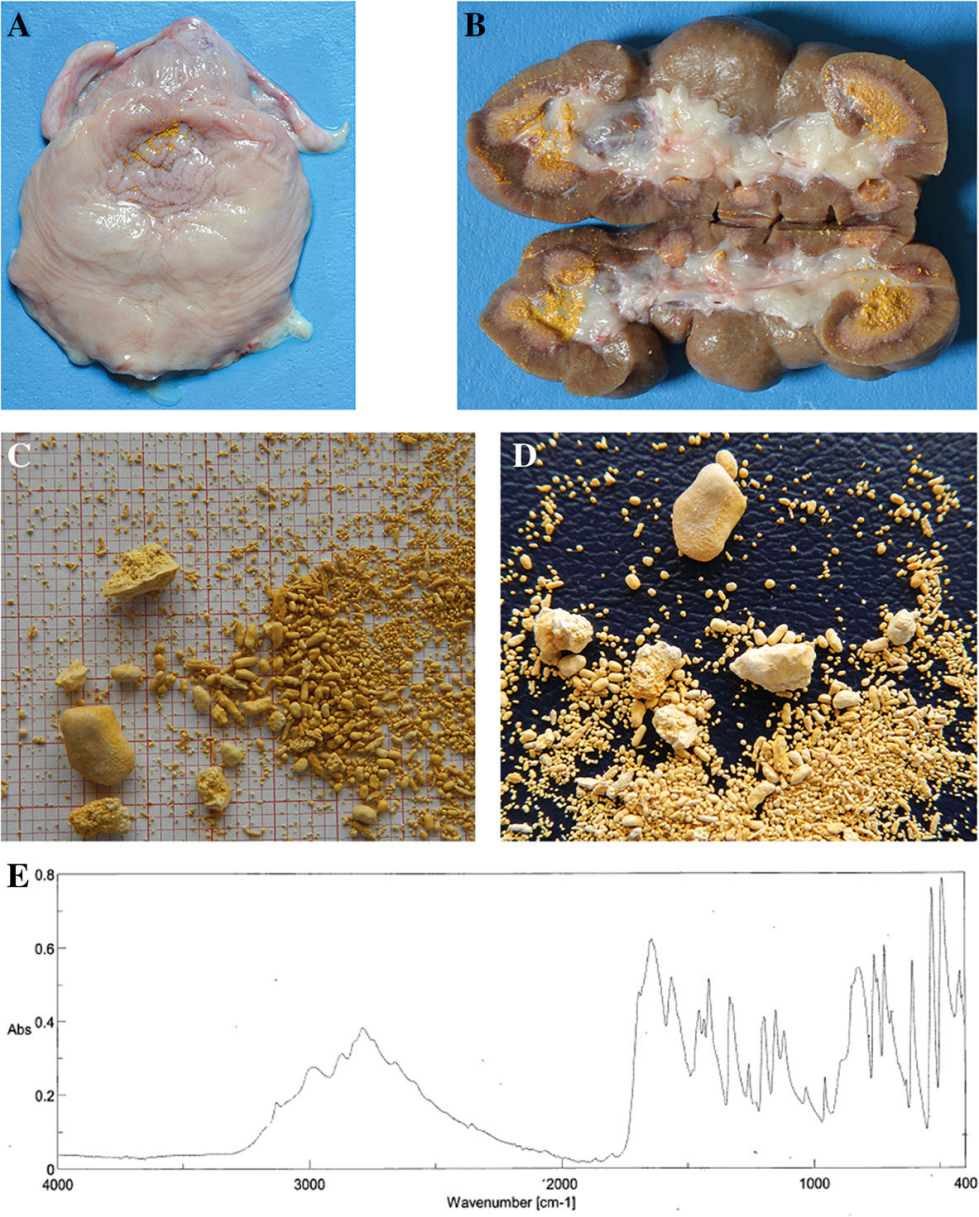
Xanthinuria in a six-month-old Tyrolean Grey heifer. Note the presence of stones visible as diffused mineralization in the granular sandy *yellow* mass in the urinary bladder (**a**) and kidney (**b**) of case 3 showing uroliths (**c**+**d**). Quantitative mineral analysis by FT-IR spectroscopy of a kidney stone demonstrated a greater than 95% xanthine content (**e**)

After the mapping and identification of the *MOCOS* variant, two novel cases were brought to the authors’ attention. Pedigree analysis demonstrated that these maternal half siblings were related to the affected twins and also showed genealogical connections to the previously identified male ancestor (Fig. 1). The animals displayed similar clinical signs as described for the affected twins [16], whereas the overgrowth of hooves was noticed only in case 3 (Fig. 4). The six-month-old heifer (case 3) was euthanized at 6 months of age and cross sections of urinary bladder and kidney indicated the presence of uroliths (Fig. 5a+b). The 21-month-old male (case 4) is still alive. Interestingly this latter animal did never show problem of hooves overgrowth, but only retarded growth and discontinuous disturbed appetite. Both affected animals were genotyped as homozygous carriers for the *MOCOS* deletion, the dam carried a single copy of the mutant allele and the two sires were known carriers, already genotyped before. In addition, we genotyped both additional cases and their dam for the missense variant in the *KIAA2026* gene. Thereby we showed that all three animals were homozygous for the wild type allele and could exclude this variant as a candidate mutation.

As the kidney stones of case 3 were freshly available, they were analyzed in order to detect their composition. The uroliths contained a combination of grit and some small oval-shaped stones (the largest measuring 8 × 5 × 3 mm). They were yellowish-brown and smooth with a glossy surface and no layers were observed (Fig. 5c+d). The quantitative mineral analysis by infrared spectroscopy using the FT-IR technique, which shows a high sensitivity and allows an accurate identification of stone composition, demonstrated a greater than 95% xanthine content (Fig. 5e).

In conclusion, the results obtained from the two additional cases confirmed the suspected xanthinuria and the disease association with the 1 bp deletion in the *MOCOS* gene.

### Role of *MOCOS* in renal conditions

Molybdenum (Mo) is present in a large number of metalloenzymes and is ubiquitous in many phyla: bacteria, archaea, fungi, algae, plants and animals [26]. Mo-containing enzymes are essential for life, holding a key position in the metabolism of the individual organism [27]. To achieve biological activity and play a role in the cell’s redox-chemistry Molybdenum must form a prosthetic group known as molybdenum cofactor (Moco) [28]. In eukaryotes, the most prominent Mo-enzymes are nitrate reductase, sulfite oxidase, and the mitochondrial amidoxime reductase, aldehyde oxidase and xanthine dehydrogenase [26]. In detail, xanthine dehydrogenase (XD) catalyzes the terminal two steps of the purine degradation pathway: formation of xanthine from hypoxanthine followed by uric acid from xanthine. This enzyme has been the focus of extensive studies for several reasons: it is a molybdenum containing flavoprotein, because of its interactions with drugs, and for its role in human disorders [29]. A deficiency of XD has been shown to cause xanthinuria which is characterized by hypouricemia and hypouricosuria [30]. Xanthinuria is classified into 2 groups, types I and II and patients with either type tend to develop urinary tract xanthine uroliths due to tissue xanthine deposits [29].

**Fig. 6:**
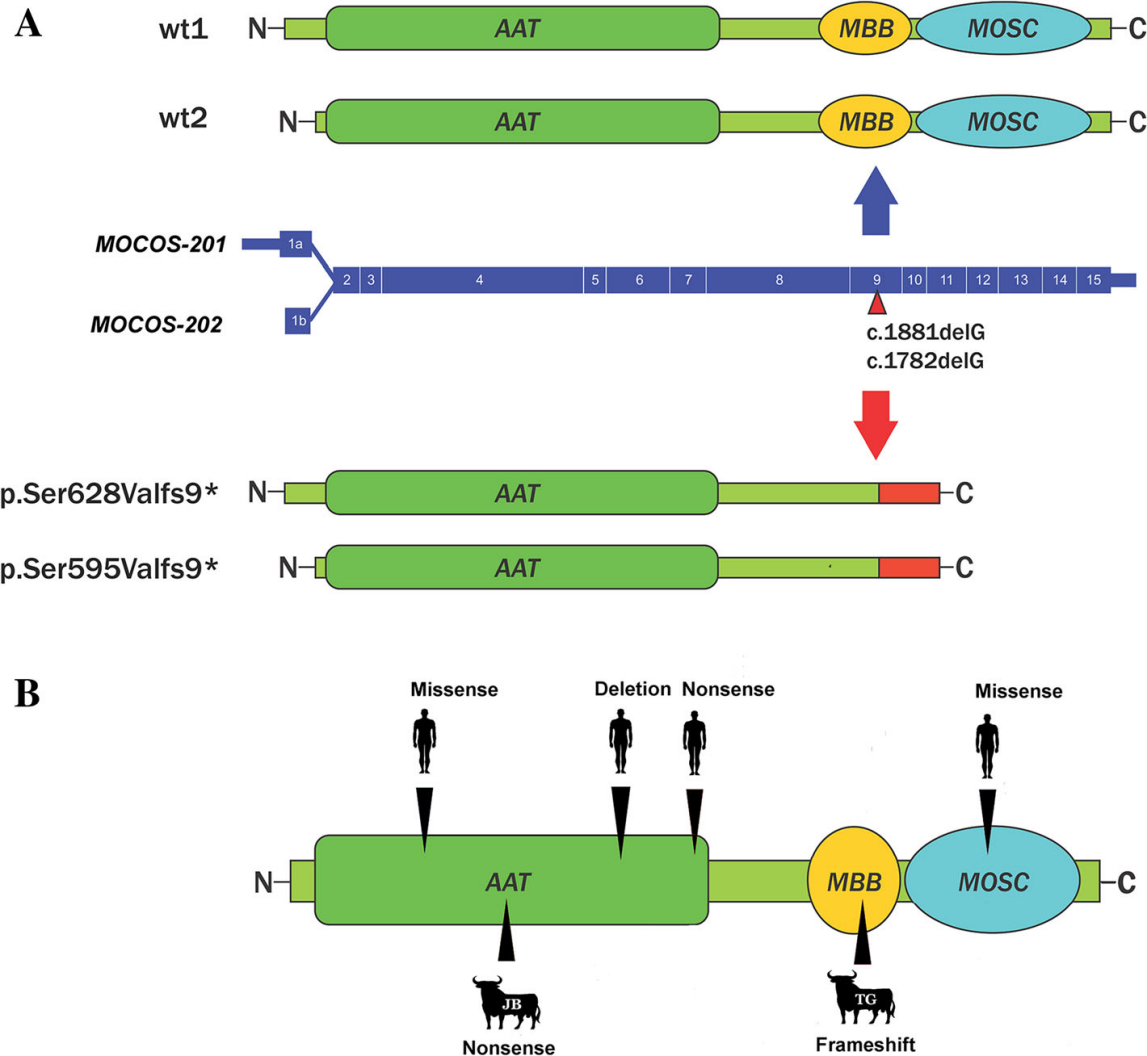
Summary of known bovine and human MOCOS mutations. **a** The two bovine *MOCOS* transcripts (*MOCOS-201* and *MOCOS-202)* are shown (*center*). The position of the mutation detected in Tyrolean Grey cattle is indicated. The two possible wild type proteins and the relevant domains (AAT: aspartate aminotransferase; MBB: mosc beta barrel; MOSC: MOSC domain) are shown above. Below, the two predicted mutant proteins are shown. Note the predicted mutant truncated proteins lack MBB and MOSC domains. **b** Previously reported mutations causing xanthinuria in human and cattle are displayed (JB: Japanese Black cattle; TG: Tyrolean Grey cattle). The nature of the predicted mutation effect of the protein is shown. The positions of mutated bovine residues are aligned to the length of the human protein

Sulfuration of the molybdenum cofactor of xanthine dehydrogenase (and aldehyde oxidase) is performed by the protein MOCOS which is required for their enzymatic activities [29]. MOCOS is therefore part of a complex pathway and the object of studies at different levels and expression studies associating the protein with genes involved in regulating transcription and apoptosis [31]. In wild silkworms, a MOCOS loss of function mutation was reported showing a mutant phenotype resulting in translucent skin [32]. In humans, mutations in *MOCOS* cause type II xanthinuria (OMIM 613274; Fig. 6). In Japanese Black cattle autosomal recessive inherited bovine xanthinuria has been reported which was characterized by elevated xanthine secretion in the urine and lethal growth retardation [14]. Affected cattle had expanded renal tubules containing xanthine calculi ranging from 1 to 3 mm in diameter. A homozygous 3 bp deletion perfectly segregated with the disease, resulting in the loss of the Tyr257 residue (p.Tyr257del) in the bovine MOCOS protein [14]. In humans, two independent patients with classical type II xanthinuria (OMIM 613274) showed a *MOCOS* c.1255C > T nonsense mutation causing a premature stop (p.Arg419*) [33]. A *MOCOS* c.466G > C missense mutation (p.Ala156Pro) was described in another xanthinuric patient [34]. Finally, a homozygous *MOCOS* c.2326C > T missense mutation (p.Arg776Cys), and a compound heterozygous with a *MOCOS* c.1034insA nonsense mutation (p.Gln347fs32*) was present in a further two xanthinuria patients [32].

In summary, cases of xanthinuria reported in the literature, caused by *MOCOS* mutations follow a recessive inheritance in both bovine and human cases. The xanthinuria occurring in Tyrolean Grey cattle reported in this study is also consistent with recessive inheritance. While it is unclear whether the mutant bovine MOCOS protein is actually expressed in our cases, with 246 amino acids or more than 30% of the normal MOCOS protein missing, it is very unlikely that a mutant protein would fulfill any physiological function. Furthermore, the mutant protein has two functionally important domains missing (Fig. 6). It is therefore more likely that the mutant mRNA is targeted by non-sense-mediated decay, thus the deleterious bovine *MOCOS* variant represents the most likely causative mutation for the renal syndrome.

In a previous case report, the authors diagnosed the renal condition as a renal dysplasia representing a developmental abnormality of the renal tissue. In defense of this diagnosis, it was inferred that the highly disruptive nature of the mutation, the severity of the condition and its probable early onset were responsible for the alteration in the structure of the developing kidney. Other macroscopical features also lead to confusion: e.g. deformity or overgrowth of hooves have been previously reported in cases of renal dysplasia in cattle [6, 10]. In papers describing xanthinuria in cattle, it was not possible to find reports of overgrown hooves [14, 15], but such features could be an indicator of a general sign of renal failure (along with poor growth) and not of a specific disease. The very limited number of cases prevents us from observing in detail how the position and specific nature of the mutation affect the onset and severity of xanthinuria. The two newly reported cases seem to show a milder phenotype compared to the initially observed affected twins. Therefore one could argue that the expressivity of the disease phenotype caused by the *MOCOS* mutation is variable.

Interestingly, there are many phenotypic differences between the initially suspected renal dysplasia and a metabolic disorder like xanthinuria. It was therefore not obvious that the identified mutation would cause the observed kidney development condition, although the detected mutation represents a deleterious loss of function mutation affecting a protein involved in renal metabolism. However, in vertebrates *MOCOS* mutations have always been associated with xanthinuria. Finally, genotyping two additional cases and the urolith analysis, confirmed the suspected diagnosis as xanthinuria.

### The third monogenic recessive defect of Tyrolean Grey cattle

Tyrolean Grey cattle (locally known in German as Grauvieh) is a very old, dual-purpose (bred for milk and meat) alpine cattle breed. The population is small with only a few thousand registered cows, predominantly in Austria (Tyrol) and Italy (South Tyrol), and fewer numbers in Switzerland. In the last decade Tyrolean Grey cattle breeders experienced outbreaks of two recessive diseases: degenerative axonopathy [19] and chondrodysplastic dwarfism [20]. For both defects, gene testing to eliminate the disease from the population is still ongoing. This report presents the breeders with a third, although obviously less frequent, genetic defect to take into consideration.

## Conclusions

This study reveals the genetic etiology of a very rare early-onset metabolic renal syndrome in Tyrolean Grey cattle. The findings allow targeted selection against a previously unknown or misdiagnosed genetic disorder affecting rearing success.

## Additional files

Additional file 1: Homozygous regions detected in the genome of the two animals with renal syndrome (xanthinuria). (PDF 30 kb)
Additional file 2: List of private homozygous sequence variants of the sequenced animal located within homozygous IBD regions. Variants with predicted effects on the protein sequence are presented boldface. (PDF 108 kb)
Additional file 3: List of private heterozygous variants in annotated genes found in the genome of the sequenced animal. (PDF 31 kb)

## Abbreviations

Bp: Base pairs
BTA: *Bos taurus* autosome
CLDN16: Claudin 16
FT-IR: Fourier transform infrared spectroscopy
Mb: Megabase pairs
MOCOS: Molybdenum cofactor sulfurase
PCR: Polymerase chain reaction
SNP: Single nucleotide polymorphism
