# Supplementary material for: A frameshift mutation in MOCOS is associated with familial renal syndrome (xanthinuria) in Tyrolean Grey cattle
Source: BMC Vet Res. 2016 Dec 5;12:276. doi: 10.1186/s12917-016-0904-4 (PMC5139135; doi:10.1186/s12917-016-0904-4)
Supplement: Additional file 1: — Homozygous regions detected in the genome of the two animals with renal syndrome (xanthinuria). (PDF 30 kb) [file 12917_2016_904_MOESM1_ESM.pdf]

Chromosome	Marker 1	Marker 2	Position 1	Position 2	Size (bp)
1	BovineHD0100000005	BovineHD0100000671	16,947	2,232,243	2,215,296
2	BovineHD0200004032	BovineHD0200006572	14,339,349	23,166,044	8,826,695
4	BovineHD0400033946	BovineHD0400035124	116,756,846	119,934,128	3,177,282
5	BovineHD0500036213	BovineHD0500029694	12,188,097	13,770,716	1,582,619
5	BovineHD0500018605	Hapmap49622-BTA-46973	66,516,549	68,294,607	1,778,058
6	BovineHD0600004525	BovineHD0600005359	16,702,620	19,302,491	2,599,871
6	Hapmap42314-BTA-75757	BovineHD0600010415	35,241,752	37,560,373	2,318,621
8	BovineHD0800002913	BovineHD0800003695	9,072,373	11,291,753	2,219,380
8	BovineHD0800031847	BovineHD0800033100	16,622,422	19,919,724	3,297,302
8	BovineHD0800010613	BovineHD0800015350	35,713,716	51,204,761	15,491,045
9	BovineHD0900009868	BovineHD0900010480	35,938,453	37,689,610	1,751,157
9	BovineHD0900015872	BovineHD0900016281	58,017,969	59,372,383	1,354,414
9	BovineHD0900023151	BovineHD0900023898	83,138,608	85,242,742	2,104,134
10	BovineHD1000014018	BovineHD1000014653	46,768,542	48,610,685	1,842,143
12	BovineHD1200006935	BovineHD1200008102	23,082,596	27,143,186	4,060,590
12	BovineHD1200020892	Hapap27863-BTA-127921	75,017,633	79,456,283	4,438,650
13	BovineHD1300002840	BovineHD1300004479	10,445,250	15,890,704	5,445,454
14	BovineHD1400012208	BovineHD1400013303	42,947,530	47,012,703	4,065,173
15	BovineHD1500010919	BovineHD1500011370	39,617,991	41,500,113	1,882,122
17	BovineHD1700007902	BovineHD1700010837	24,929,262	39,788,696	14,859,434
17	BovineHD1700014854	BovineHD4100013206	52,764,123	60,673,836	7,909,713
18	BovineHD1800005514	BovineHD1800006053	10,852,558	19,489,001	8,636,443
19	BovineHD1900004545	ARS-BFGL-NGS-54750	16,477,155	18,775,014	2,297,859
19	BovineHD1900017278	BovineHD1900018194	60,358,453	62,991,496	2,633,043
21	BovineHD2100015375	BovineHD2100016166	53,731,018	56,274,431	2,543,413
23	BovineHD2300002663	BovineHD2300003693	10,851,148	14,657,263	3,806,115
24	BovineHD2400000060	BovineHD2400001376	392,019	4,963,234	4,571,215
24	BovineHD2400007408	BovineHD2400008718	21,002,290	32,030,629	11,028,339
24	BovineHD4100016625	BovineHD2400011397	39,372,536	41,143,699	1,771,163
24	BovineHD2400015944	BovineHD2400016771	55,829,656	58,346,014	2,516,358
27	BovineHD2700009302	BovineHD2700010132	32,923,106	35,558,822	2,635,716

Additional file 1:

Homozygous regions detected in the genome of the two animals with renal syndrome.
