# Supplementary material for: A frameshift mutation in MOCOS is associated with familial renal syndrome (xanthinuria) in Tyrolean Grey cattle
Source: BMC Vet Res. 2016 Dec 5;12:276. doi: 10.1186/s12917-016-0904-4 (PMC5139135; doi:10.1186/s12917-016-0904-4)
Supplement: Additional file 2: — List of private homozygous sequence variants of the sequenced animal located within homozygous IBD regions. Variants with predicted effects on the protein sequence are presented boldface. (PDF 108 kb) [file 12917_2016_904_MOESM2_ESM.pdf]

Additional file 2:

List of private homozygous sequence variants of the sequenced animal located within homozygous IBD regions. Variants with predicted effects on the protein sequence are presented boldface.

Chromosome	*Position*	*REF*	*ALT*	*EFFECT*	*IMPACT*	*CODON*	*GENE*	*BIOTYPE*	*TRID*
4	117598089	T	TC	intron_variant	MODIFIER	c.375-5405_375-5404insC	DPP6	protein_coding	ENSBTAT00000029251
4	119134084	C	T	intron_variant	MODIFIER	c.967-458C>T	NOM1	protein_coding	ENSBTAT00000043572
6	16743580	TGGA	T	upstream_gene_variant	MODIFIER	c.-2888_-2886delTCC	RRH	protein_coding	ENSBTAT00000000997
6	16743580	TGGA	T	downstream_gene_variant	MODIFIER	c.*4440_*4442delTCC	GAR1	protein_coding	ENSBTAT00000000992
6	16823750	C	CTATG	upstream_gene_variant	MODIFIER	c.-4986_-4985insTATG	PLA2G12A	protein_coding	ENSBTAT00000020263
6	16919900	C	T	intron_variant	MODIFIER	c.97-28741G>A	CCDC109B	protein_coding	ENSBTAT00000017276
6	17826103	G	A	downstream_gene_variant	MODIFIER	c.*2047C>T	RPL34	protein_coding	ENSBTAT00000018910
6	17828429	ATTAAT	A	intron_variant	MODIFIER	c.303-200_303-196delATTAA	RPL34	protein_coding	ENSBTAT00000018910
6	17829011	T	C	intron_variant	MODIFIER	c.303-777A>G	RPL34	protein_coding	ENSBTAT00000018910
6	17829229	T	TC	intron_variant	MODIFIER	c.303-996_303-995insG	RPL34	protein_coding	ENSBTAT00000018910
6	17829935	C	A	intron_variant	MODIFIER	c.302+1182G>T	RPL34	protein_coding	ENSBTAT00000018910
6	17834638	G	A	upstream_gene_variant	MODIFIER	c.-1927C>T	RPL34	protein_coding	ENSBTAT00000018910
6	17837169	G	A	upstream_gene_variant	MODIFIER	c.-4458C>T	RPL34	protein_coding	ENSBTAT00000018910
6	18386511	C	T	intron_variant	MODIFIER	c.415-25580C>T	LEF1	protein_coding	ENSBTAT00000008991
8	36597460	G	A	intron_variant	MODIFIER	c.1160-2921G>A	PTPRD	protein_coding	ENSBTAT00000013433
8	36795063	C	G	intron_variant	MODIFIER	c.5127-103C>G	PTPRD	protein_coding	ENSBTAT00000013433
8	38555403	C	T	intron_variant	MODIFIER	c.2838+490C>T	GLDC	protein_coding	ENSBTAT00000036700
8	38591977	G	A	intron_variant	MODIFIER	c.1013+1114C>T	UHRF2	protein_coding	ENSBTAT00000027737
8	38687001	T	A	upstream_gene_variant	MODIFIER	n.-2885A>T	ENSBTAG00000011161	processed_pseudogene	ENSBTAT00000014822
8	38687001	T	A	downstream_gene_variant	MODIFIER	c.*4667A>T	TPD52L3	protein_coding	ENSBTAT00000014821
8	38744726	A	C	intron_variant	MODIFIER	c.-11-296T>G	IL33	protein_coding	ENSBTAT00000024412
8	38754204	A	G	intron_variant	MODIFIER	c.-167-6891T>C	IL33	protein_coding	ENSBTAT00000024412
8	38754215	G	A	intron_variant	MODIFIER	c.-167-6902C>T	IL33	protein_coding	ENSBTAT00000024412
8	38793421	G	A	upstream_gene_variant	MODIFIER	c.-49002C>T	IL33	protein_coding	ENSBTAT00000024412
8	38922727	C	T	upstream_gene_variant	MODIFIER	c.-180C>T	RANBP6	protein_coding	ENSBTAT00000016139
8	38929216	G	A	downstream_gene_variant	MODIFIER	c.*2992G>A	RANBP6	protein_coding	ENSBTAT00000016139
**8**	**39038055**	**C**	**G**	**missense_variant**	**MODERATE**	**c.926C>G**	**KIAA2026**	**protein_coding**	**ENSBTAT00000063356**
8	39039595	A	C	downstream_gene_variant	MODIFIER	c.*699A>C	KIAA2026	protein_coding	ENSBTAT00000063356
8	39040902	T	C	downstream_gene_variant	MODIFIER	c.*2006T>C	KIAA2026	protein_coding	ENSBTAT00000063356
8	39043867	C	T	downstream_gene_variant	MODIFIER	c.*4971C>T	KIAA2026	protein_coding	ENSBTAT00000063356
8	39052947	C	T	intron_variant	MODIFIER	c.175-693G>A	MLANA	protein_coding	ENSBTAT00000009784
8	39053495	C	T	intron_variant	MODIFIER	c.175-1241G>A	MLANA	protein_coding	ENSBTAT00000009784
8	39198684	T	G	intron_variant	MODIFIER	c.3614-757A>C	RIC1	protein_coding	ENSBTAT00000001692
8	39518556	A	C	upstream_gene_variant	MODIFIER	c.-8302T>G	PD-L1	protein_coding	ENSBTAT00000008067
8	40251861	G	A	intron_variant	MODIFIER	c.91+1156C>T	SLC1A1	protein_coding	ENSBTAT00000025456
8	40252778	G	A	intron_variant	MODIFIER	c.91+239C>T	SLC1A1	protein_coding	ENSBTAT00000025456
8	42823339	T	C	intron_variant	MODIFIER	c.-36-4330A>G	SMARCA2	protein_coding	ENSBTAT00000035681
8	44544016	C	T	intron_variant	MODIFIER	c.53+1270G>A	DOCK8	protein_coding	ENSBTAT00000002840
8	45214924	G	A	upstream_gene_variant	MODIFIER	c.-55404G>A	PIP5K1B	protein_coding	ENSBTAT00000036688
8	45346048	A	G	downstream_gene_variant	MODIFIER	n.*3487T>C	snoU13	snoRNA	ENSBTAT00000062326
8	45346048	A	G	intron_variant	MODIFIER	c.1133-3181A>G	PIP5K1B	protein_coding	ENSBTAT00000036688
8	45486980	A	G	upstream_gene_variant	MODIFIER	n.-4873A>G	5S_rRNA	rRNA	ENSBTAT00000062666
8	45487146	G	C	upstream_gene_variant	MODIFIER	n.-4707G>C	5S_rRNA	rRNA	ENSBTAT00000062666
8	45487179	G	A	upstream_gene_variant	MODIFIER	n.-4674G>A	5S_rRNA	rRNA	ENSBTAT00000062666
8	45487597	G	C	upstream_gene_variant	MODIFIER	n.-4256G>C	5S_rRNA	rRNA	ENSBTAT00000062666
8	45488763	G	A	upstream_gene_variant	MODIFIER	n.-3090G>A	5S_rRNA	rRNA	ENSBTAT00000062666
8	45488904	C	T	upstream_gene_variant	MODIFIER	n.-2949C>T	5S_rRNA	rRNA	ENSBTAT00000062666
8	45488969	C	T	upstream_gene_variant	MODIFIER	n.-2884C>T	5S_rRNA	rRNA	ENSBTAT00000062666
8	45489561	T	G	upstream_gene_variant	MODIFIER	n.-2292T>G	5S_rRNA	rRNA	ENSBTAT00000062666
8	45489698	G	A	upstream_gene_variant	MODIFIER	n.-2155G>A	5S_rRNA	rRNA	ENSBTAT00000062666
8	45489803	G	A	upstream_gene_variant	MODIFIER	n.-2050G>A	5S_rRNA	rRNA	ENSBTAT00000062666
8	45489842	G	A	upstream_gene_variant	MODIFIER	n.-2011G>A	5S_rRNA	rRNA	ENSBTAT00000062666
8	45490008	G	T	upstream_gene_variant	MODIFIER	n.-1845G>T	5S_rRNA	rRNA	ENSBTAT00000062666
8	45490195	A	C	upstream_gene_variant	MODIFIER	n.-1658A>C	5S_rRNA	rRNA	ENSBTAT00000062666
8	45490197	A	G	upstream_gene_variant	MODIFIER	n.-1656A>G	5S_rRNA	rRNA	ENSBTAT00000062666
8	45490756	A	T	upstream_gene_variant	MODIFIER	n.-1097A>T	5S_rRNA	rRNA	ENSBTAT00000062666
8	45491283	AAG	A	upstream_gene_variant	MODIFIER	n.-569_-568delAG	5S_rRNA	rRNA	ENSBTAT00000062666
8	45491287	T	A	upstream_gene_variant	MODIFIER	n.-566T>A	5S_rRNA	rRNA	ENSBTAT00000062666
8	45491306	C	T	upstream_gene_variant	MODIFIER	n.-547C>T	5S_rRNA	rRNA	ENSBTAT00000062666
8	45492214	C	T	downstream_gene_variant	MODIFIER	n.*269C>T	5S_rRNA	rRNA	ENSBTAT00000062666
8	45492951	T	C	downstream_gene_variant	MODIFIER	n.*1006T>C	5S_rRNA	rRNA	ENSBTAT00000062666
8	45493363	T	G	downstream_gene_variant	MODIFIER	n.*1418T>G	5S_rRNA	rRNA	ENSBTAT00000062666
8	45493378	G	A	downstream_gene_variant	MODIFIER	n.*1433G>A	5S_rRNA	rRNA	ENSBTAT00000062666
8	45493436	A	G	downstream_gene_variant	MODIFIER	n.*1491A>G	5S_rRNA	rRNA	ENSBTAT00000062666
8	45493720	A	C	downstream_gene_variant	MODIFIER	n.*1775A>C	5S_rRNA	rRNA	ENSBTAT00000062666
8	45494116	T	C	downstream_gene_variant	MODIFIER	n.*2171T>C	5S_rRNA	rRNA	ENSBTAT00000062666
8	45494191	C	G	downstream_gene_variant	MODIFIER	n.*2246C>G	5S_rRNA	rRNA	ENSBTAT00000062666
8	45494197	G	A	downstream_gene_variant	MODIFIER	n.*2252G>A	5S_rRNA	rRNA	ENSBTAT00000062666
8	45494347	C	T	downstream_gene_variant	MODIFIER	n.*2402C>T	5S_rRNA	rRNA	ENSBTAT00000062666
8	45494404	A	G	downstream_gene_variant	MODIFIER	n.*2459A>G	5S_rRNA	rRNA	ENSBTAT00000062666
8	45494522	T	C	downstream_gene_variant	MODIFIER	n.*2577T>C	5S_rRNA	rRNA	ENSBTAT00000062666
8	45494545	G	A	downstream_gene_variant	MODIFIER	n.*2600G>A	5S_rRNA	rRNA	ENSBTAT00000062666
8	45495136	G	A	downstream_gene_variant	MODIFIER	n.*3191G>A	5S_rRNA	rRNA	ENSBTAT00000062666
8	45495177	C	T	downstream_gene_variant	MODIFIER	n.*3232C>T	5S_rRNA	rRNA	ENSBTAT00000062666
8	45495679	T	C	downstream_gene_variant	MODIFIER	n.*3734T>C	5S_rRNA	rRNA	ENSBTAT00000062666
8	45496266	A	C	downstream_gene_variant	MODIFIER	n.*4321A>C	5S_rRNA	rRNA	ENSBTAT00000062666
8	45496347	T	C	downstream_gene_variant	MODIFIER	n.*4402T>C	5S_rRNA	rRNA	ENSBTAT00000062666
8	45496486	C	T	downstream_gene_variant	MODIFIER	n.*4541C>T	5S_rRNA	rRNA	ENSBTAT00000062666
8	45496532	G	A	downstream_gene_variant	MODIFIER	n.*4587G>A	5S_rRNA	rRNA	ENSBTAT00000062666
8	45496603	T	C	downstream_gene_variant	MODIFIER	n.*4658T>C	5S_rRNA	rRNA	ENSBTAT00000062666
8	45496770	G	A	downstream_gene_variant	MODIFIER	n.*4825G>A	5S_rRNA	rRNA	ENSBTAT00000062666
8	45496785	A	G	downstream_gene_variant	MODIFIER	n.*4840A>G	5S_rRNA	rRNA	ENSBTAT00000062666
8	45499073	G	A	upstream_gene_variant	MODIFIER	c.-4928G>A	FXN	protein_coding	ENSBTAT00000001725
8	45499525	T	C	upstream_gene_variant	MODIFIER	c.-4476T>C	FXN	protein_coding	ENSBTAT00000001725
8	45503463	A	G	upstream_gene_variant	MODIFIER	c.-538A>G	FXN	protein_coding	ENSBTAT00000001725
8	45503482	A	G	upstream_gene_variant	MODIFIER	c.-519A>G	FXN	protein_coding	ENSBTAT00000001725
8	45503499	C	G	upstream_gene_variant	MODIFIER	c.-502C>G	FXN	protein_coding	ENSBTAT00000001725
8	45503952	T	C	upstream_gene_variant	MODIFIER	c.-49T>C	FXN	protein_coding	ENSBTAT00000001725
8	45503984	C	T	5_prime_UTR_premature_start_codon_gain_variant	LOW	c.-17C>T	FXN	protein_coding	ENSBTAT00000001725
8	45503984	C	T	5_prime_UTR_variant	MODIFIER	c.-17C>T	FXN	protein_coding	ENSBTAT00000001725
8	45504858	T	C	intron_variant	MODIFIER	c.168+690T>C	FXN	protein_coding	ENSBTAT00000001725
8	45504871	A	C	intron_variant	MODIFIER	c.168+703A>C	FXN	protein_coding	ENSBTAT00000001725
8	45505013	A	G	intron_variant	MODIFIER	c.168+845A>G	FXN	protein_coding	ENSBTAT00000001725
8	45505089	C	T	intron_variant	MODIFIER	c.168+921C>T	FXN	protein_coding	ENSBTAT00000001725
8	45505493	C	T	intron_variant	MODIFIER	c.168+1325C>T	FXN	protein_coding	ENSBTAT00000001725
8	45505494	A	G	intron_variant	MODIFIER	c.168+1326A>G	FXN	protein_coding	ENSBTAT00000001725
8	45505524	A	C	intron_variant	MODIFIER	c.168+1356A>C	FXN	protein_coding	ENSBTAT00000001725
8	45505527	T	C	intron_variant	MODIFIER	c.168+1359T>C	FXN	protein_coding	ENSBTAT00000001725
8	45505557	T	G	intron_variant	MODIFIER	c.168+1389T>G	FXN	protein_coding	ENSBTAT00000001725
8	45505558	CG	C	intron_variant	MODIFIER	c.168+1391delG	FXN	protein_coding	ENSBTAT00000001725
8	45505598	A	G	intron_variant	MODIFIER	c.168+1430A>G	FXN	protein_coding	ENSBTAT00000001725
8	45505697	C	T	intron_variant	MODIFIER	c.168+1529C>T	FXN	protein_coding	ENSBTAT00000001725
8	45505703	C	T	intron_variant	MODIFIER	c.168+1535C>T	FXN	protein_coding	ENSBTAT00000001725
8	45505726	A	G	intron_variant	MODIFIER	c.168+1558A>G	FXN	protein_coding	ENSBTAT00000001725
8	45505775	G	A	intron_variant	MODIFIER	c.168+1607G>A	FXN	protein_coding	ENSBTAT00000001725
8	45505828	GC	G	intron_variant	MODIFIER	c.168+1661delC	FXN	protein_coding	ENSBTAT00000001725
8	45505832	C	T	intron_variant	MODIFIER	c.168+1664C>T	FXN	protein_coding	ENSBTAT00000001725
8	45505840	T	C	intron_variant	MODIFIER	c.168+1672T>C	FXN	protein_coding	ENSBTAT00000001725
8	45505842	A	G	intron_variant	MODIFIER	c.168+1674A>G	FXN	protein_coding	ENSBTAT00000001725

8	45505849	C	A	intron_variant	MODIFIER	c.168+1681C>A	FXN	protein_coding	ENSBTAT00000001725
8	45505852	T	A	intron_variant	MODIFIER	c.168+1684T>A	FXN	protein_coding	ENSBTAT00000001725
8	45505857	T	C	intron_variant	MODIFIER	c.168+1689T>C	FXN	protein_coding	ENSBTAT00000001725
8	45505924	A	G	intron_variant	MODIFIER	c.168+1756A>G	FXN	protein_coding	ENSBTAT00000001725
8	45505935	G	C	intron_variant	MODIFIER	c.168+1767G>C	FXN	protein_coding	ENSBTAT00000001725
8	45505936	A	AT	intron_variant	MODIFIER	c.168+1768_168+1769insT	FXN	protein_coding	ENSBTAT00000001725
8	45505955	C	A	intron_variant	MODIFIER	c.168+1787C>A	FXN	protein_coding	ENSBTAT00000001725
8	45505980	T	C	intron_variant	MODIFIER	c.168+1812T>C	FXN	protein_coding	ENSBTAT00000001725
8	45506047	T	G	intron_variant	MODIFIER	c.168+1879T>G	FXN	protein_coding	ENSBTAT00000001725
8	45506251	C	T	intron_variant	MODIFIER	c.168+2083C>T	FXN	protein_coding	ENSBTAT00000001725
8	45506707	C	T	intron_variant	MODIFIER	c.168+2539C>T	FXN	protein_coding	ENSBTAT00000001725
8	45506743	G	C	intron_variant	MODIFIER	c.168+2575G>C	FXN	protein_coding	ENSBTAT00000001725
8	45507034	C	T	intron_variant	MODIFIER	c.169-2297C>T	FXN	protein_coding	ENSBTAT00000001725
8	45507145	C	T	intron_variant	MODIFIER	c.169-2186C>T	FXN	protein_coding	ENSBTAT00000001725
8	45507277	T	C	intron_variant	MODIFIER	c.169-2054T>C	FXN	protein_coding	ENSBTAT00000001725
8	45507459	G	T	intron_variant	MODIFIER	c.169-1872G>T	FXN	protein_coding	ENSBTAT00000001725
8	45507524	C	T	intron_variant	MODIFIER	c.169-1807C>T	FXN	protein_coding	ENSBTAT00000001725
8	45507695	A	G	intron_variant	MODIFIER	c.169-1636A>G	FXN	protein_coding	ENSBTAT00000001725
8	45507715	T	C	intron_variant	MODIFIER	c.169-1616T>C	FXN	protein_coding	ENSBTAT00000001725
8	45507894	T	G	intron_variant	MODIFIER	c.169-1437T>G	FXN	protein_coding	ENSBTAT00000001725
8	45509017	A	G	intron_variant	MODIFIER	c.169-314A>G	FXN	protein_coding	ENSBTAT00000001725
8	45509018	C	T	intron_variant	MODIFIER	c.169-313C>T	FXN	protein_coding	ENSBTAT00000001725
8	45510634	C	A	intron_variant	MODIFIER	c.266+1206C>A	FXN	protein_coding	ENSBTAT00000001725
8	45510648	C	T	intron_variant	MODIFIER	c.266+1220C>T	FXN	protein_coding	ENSBTAT00000001725
8	45510728	C	T	intron_variant	MODIFIER	c.266+1300C>T	FXN	protein_coding	ENSBTAT00000001725
8	45512468	G	C	intron_variant	MODIFIER	c.267-2206G>C	FXN	protein_coding	ENSBTAT00000001725
8	45514964	G	C	intron_variant	MODIFIER	c.387+170G>C	FXN	protein_coding	ENSBTAT00000001725
8	45516225	T	C	intron_variant	MODIFIER	c.387+1431T>C	FXN	protein_coding	ENSBTAT00000001725
8	45517774	G	A	intron_variant	MODIFIER	c.387+2980G>A	FXN	protein_coding	ENSBTAT00000001725
8	45519408	G	A	intron_variant	MODIFIER	c.388-1815G>A	FXN	protein_coding	ENSBTAT00000001725
8	45519633	G	A	intron_variant	MODIFIER	c.388-1590G>A	FXN	protein_coding	ENSBTAT00000001725
8	45528856	T	C	downstream_gene_variant	MODIFIER	c.*1356T>C	FXN	protein_coding	ENSBTAT00000001725
8	45878592	G	A	intron_variant	MODIFIER	c.2175+1225C>T	APBA1	protein_coding	ENSBTAT00000011688
8	46635751	A	C	intron_variant	MODIFIER	c.1019-2060A>C	MAMDC2	protein_coding	ENSBTAT00000023092
8	46686651	T	C	intron_variant	MODIFIER	c.1792-2570T>C	MAMDC2	protein_coding	ENSBTAT00000023092
8	47051771	G	T	intron_variant	MODIFIER	c.3133+20382C>A	TRPM3	protein_coding	ENSBTAT00000061517
8	48435328	A	C	intron_variant	MODIFIER	c.-3-19258T>G	FAM108B1	protein_coding	ENSBTAT00000008957
8	48453730	C	G	upstream_gene_variant	MODIFIER	c.-2680C>G	C8H9ORF85	protein_coding	ENSBTAT00000008975
8	48453730	C	G	intron_variant	MODIFIER	c.-4+1578G>C	FAM108B1	protein_coding	ENSBTAT00000008957
8	48481515	C	CA	intron_variant	MODIFIER	c.103-9678_103-9677insA	C8H9ORF85	protein_coding	ENSBTAT00000008975
8	48701986	G	C	intron_variant	MODIFIER	c.124-27302G>C	GDA	protein_coding	ENSBTAT00000015339
8	48762541	CA	C	intron_variant	MODIFIER	c.988+3212delA	GDA	protein_coding	ENSBTAT00000015339
9	36713660	T	C	intron_variant	MODIFIER	c.-145-14269T>C	HS3ST5	protein_coding	ENSBTAT00000000213
9	36774504	G	A	intron_variant	MODIFIER	c.-33+46463G>A	HS3ST5	protein_coding	ENSBTAT00000000213
9	36775751	G	C	intron_variant	MODIFIER	c.-33+47710G>C	HS3ST5	protein_coding	ENSBTAT00000000213
9	36779755	TC	T	intron_variant	MODIFIER	c.-33+51715delC	HS3ST5	protein_coding	ENSBTAT00000000213
9	36792378	C	A	intron_variant	MODIFIER	c.-32-46312C>A	HS3ST5	protein_coding	ENSBTAT00000000213
9	36793282	G	A	intron_variant	MODIFIER	c.-32-45408G>A	HS3ST5	protein_coding	ENSBTAT00000000213
9	36793689	A	C	intron_variant	MODIFIER	c.-32-45001A>C	HS3ST5	protein_coding	ENSBTAT00000000213
10	47533504	A	G	intron_variant	MODIFIER	c.-36-19403T>C	TLN2	protein_coding	ENSBTAT00000061376
10	47961801	CAT	C	downstream_gene_variant	MODIFIER	c.*4412_*4413delAT	C2CD4A	protein_coding	ENSBTAT00000027736
10	48248855	CTG	C	intron_variant	MODIFIER	c.10012-144_10012-143delTG	VPS13C	protein_coding	ENSBTAT00000061287
12	24156210	C	G	intron_variant	MODIFIER	c.1241-5105C>G	TRPC4	protein_coding	ENSBTAT00000012378
12	78059230	C	T	upstream_gene_variant	MODIFIER	c.-4614C>T	OXGR1	protein_coding	ENSBTAT00000035454
12	79380842	C	T	intron_variant	MODIFIER	c.1417+5538C>T	ENSBTAG00000000146	protein_coding	ENSBTAT00000000167
12	79380887	C	T	intron_variant	MODIFIER	c.1417+5583C>T	ENSBTAG00000000146	protein_coding	ENSBTAT00000000167
12	79381341	C	T	intron_variant	MODIFIER	c.1417+6037C>T	ENSBTAG00000000146	protein_coding	ENSBTAT00000000167
13	10945513	A	G	intron_variant	MODIFIER	c.280+2886A>G	ENSBTAG00000023169	protein_coding	ENSBTAT00000050711
13	10955908	C	A	intron_variant	MODIFIER	c.865-591C>A	ENSBTAG00000023169	protein_coding	ENSBTAT00000050711
13	10968546	G	A	intron_variant	MODIFIER	c.1678-677G>A	ENSBTAG00000023169	protein_coding	ENSBTAT00000050711
13	11329141	G	C	intron_variant	MODIFIER	c.706-605G>C	ANKRD26	protein_coding	ENSBTAT00000031151
13	11329141	G	C	intron_variant	MODIFIER	c.299+122G>C	ANKRD26	protein_coding	ENSBTAT00000055503
13	12298609	TA	T	intron_variant	MODIFIER	c.2323-470delT	DHTKD1	protein_coding	ENSBTAT00000000364
14	44970900	C	T	intron_variant	MODIFIER	c.19+1776C>T	STMN2	protein_coding	ENSBTAT00000026776
14	45297375	T	C	intron_variant	MODIFIER	c.399-17785A>G	MRPS28	protein_coding	ENSBTAT00000001718
14	45352499	G	C	intron_variant	MODIFIER	c.399-72909C>G	MRPS28	protein_coding	ENSBTAT00000001718
14	45413670	G	A	intron_variant	MODIFIER	c.398+61892C>T	MRPS28	protein_coding	ENSBTAT00000001718
14	45442660	C	T	intron_variant	MODIFIER	c.398+32902G>A	MRPS28	protein_coding	ENSBTAT00000001718
14	45504865	C	A	intron_variant	MODIFIER	c.624+312G>T	TPD52	protein_coding	ENSBTAT00000055321
14	45504865	C	A	intron_variant	MODIFIER	c.435+312G>T	TPD52	protein_coding	ENSBTAT00000054316
14	45504865	C	A	intron_variant	MODIFIER	c.555+312G>T	TPD52	protein_coding	ENSBTAT00000037262
14	45830553	T	A	intron_variant	MODIFIER	c.966+1659T>A	ZBTB10	protein_coding	ENSBTAT00000004333
14	45830774	T	TC	intron_variant	MODIFIER	c.966+1880_966+1881insC	ZBTB10	protein_coding	ENSBTAT00000004333
14	45992939	C	T	downstream_gene_variant	MODIFIER	c.*2438G>A	ZNF704	protein_coding	ENSBTAT00000028982
14	45995373	C	A	3_prime_UTR_variant	MODIFIER	c.*4G>T	ZNF704	protein_coding	ENSBTAT00000028982
14	45995671	A	G	intron_variant	MODIFIER	c.915-183T>C	ZNF704	protein_coding	ENSBTAT00000028982
14	45995787	A	T	intron_variant	MODIFIER	c.915-299T>A	ZNF704	protein_coding	ENSBTAT00000028982
14	45996342	G	A	intron_variant	MODIFIER	c.914+121C>T	ZNF704	protein_coding	ENSBTAT00000028982
14	45997060	G	A	intron_variant	MODIFIER	c.817-500C>T	ZNF704	protein_coding	ENSBTAT00000028982
14	45998126	T	C	intron_variant	MODIFIER	c.817-1566A>G	ZNF704	protein_coding	ENSBTAT00000028982
14	46000274	C	T	intron_variant	MODIFIER	c.817-3714G>A	ZNF704	protein_coding	ENSBTAT00000028982
14	46002299	C	G	intron_variant	MODIFIER	c.816+4874G>C	ZNF704	protein_coding	ENSBTAT00000028982
14	46007734	A	T	intron_variant	MODIFIER	c.712-457T>A	ZNF704	protein_coding	ENSBTAT00000028982
14	46008281	A	T	intron_variant	MODIFIER	c.712-1004T>A	ZNF704	protein_coding	ENSBTAT00000028982
14	46008596	C	T	intron_variant	MODIFIER	c.711+934G>A	ZNF704	protein_coding	ENSBTAT00000028982
14	46009058	C	T	intron_variant	MODIFIER	c.711+472G>A	ZNF704	protein_coding	ENSBTAT00000028982
14	46009479	C	T	intron_variant	MODIFIER	c.711+51G>A	ZNF704	protein_coding	ENSBTAT00000028982
14	46009512	T	C	intron_variant	MODIFIER	c.711+18A>G	ZNF704	protein_coding	ENSBTAT00000028982
14	46009656	G	A	synonymous_variant	LOW	c.585C>T	ZNF704	protein_coding	ENSBTAT00000028982
14	46010003	T	C	intron_variant	MODIFIER	c.444-206A>G	ZNF704	protein_coding	ENSBTAT00000028982
14	46010099	G	A	intron_variant	MODIFIER	c.444-302C>T	ZNF704	protein_coding	ENSBTAT00000028982
14	46010294	GA	G	intron_variant	MODIFIER	c.444-498delT	ZNF704	protein_coding	ENSBTAT00000028982
14	46010367	C	T	intron_variant	MODIFIER	c.444-570G>A	ZNF704	protein_coding	ENSBTAT00000028982
14	46010424	A	T	intron_variant	MODIFIER	c.444-627T>A	ZNF704	protein_coding	ENSBTAT00000028982
14	46010710	A	G	intron_variant	MODIFIER	c.444-913T>C	ZNF704	protein_coding	ENSBTAT00000028982
14	46010716	C	T	intron_variant	MODIFIER	c.444-919G>A	ZNF704	protein_coding	ENSBTAT00000028982
14	46010717	A	G	intron_variant	MODIFIER	c.444-920T>C	ZNF704	protein_coding	ENSBTAT00000028982
14	46010795	A	T	intron_variant	MODIFIER	c.444-998T>A	ZNF704	protein_coding	ENSBTAT00000028982
14	46010802	GA	G	intron_variant	MODIFIER	c.444-1006delT	ZNF704	protein_coding	ENSBTAT00000028982
14	46010832	G	A	intron_variant	MODIFIER	c.444-1035C>T	ZNF704	protein_coding	ENSBTAT00000028982
14	46010961	A	C	intron_variant	MODIFIER	c.444-1164T>G	ZNF704	protein_coding	ENSBTAT00000028982
14	46011199	A	C	intron_variant	MODIFIER	c.444-1402T>G	ZNF704	protein_coding	ENSBTAT00000028982
14	46011426	T	C	intron_variant	MODIFIER	c.444-1629A>G	ZNF704	protein_coding	ENSBTAT00000028982
14	46011521	T	G	intron_variant	MODIFIER	c.444-1724A>C	ZNF704	protein_coding	ENSBTAT00000028982
14	46011763	G	A	intron_variant	MODIFIER	c.444-1966C>T	ZNF704	protein_coding	ENSBTAT00000028982
14	46013672	A	T	intron_variant	MODIFIER	c.444-3875T>A	ZNF704	protein_coding	ENSBTAT00000028982
14	46013738	G	A	intron_variant	MODIFIER	c.444-3941C>T	ZNF704	protein_coding	ENSBTAT00000028982
14	46016920	G	A	intron_variant	MODIFIER	c.443+1801C>T	ZNF704	protein_coding	ENSBTAT00000028982
14	46020537	C	T	intron_variant	MODIFIER	c.343-1716G>A	ZNF704	protein_coding	ENSBTAT00000028982
14	46021000	T	G	intron_variant	MODIFIER	c.343-2179A>C	ZNF704	protein_coding	ENSBTAT00000028982
14	46021460	C	T	intron_variant	MODIFIER	c.343-2639G>A	ZNF704	protein_coding	ENSBTAT00000028982
14	46021525	T	C	intron_variant	MODIFIER	c.343-2704A>G	ZNF704	protein_coding	ENSBTAT00000028982
14	46022330	A	C	intron_variant	MODIFIER	c.343-3509T>G	ZNF704	protein_coding	ENSBTAT00000028982

14	46022818	A	G	intron_variant	MODIFIER	c.343-3997T>C	ZNF704	protein_coding	ENSBTAT00000028982
14	46025661	C	A	intron_variant	MODIFIER	c.343-6840G>T	ZNF704	protein_coding	ENSBTAT00000028982
14	46026718	G	A	intron_variant	MODIFIER	c.343-7897C>T	ZNF704	protein_coding	ENSBTAT00000028982
14	46026741	G	C	intron_variant	MODIFIER	c.343-7920C>G	ZNF704	protein_coding	ENSBTAT00000028982
14	46026862	CT	C	intron_variant	MODIFIER	c.343-8042delA	ZNF704	protein_coding	ENSBTAT00000028982
14	46027620	G	A	intron_variant	MODIFIER	c.342+7485C>T	ZNF704	protein_coding	ENSBTAT00000028982
14	46027637	C	T	intron_variant	MODIFIER	c.342+7468G>A	ZNF704	protein_coding	ENSBTAT00000028982
14	46027638	G	A	intron_variant	MODIFIER	c.342+7467C>T	ZNF704	protein_coding	ENSBTAT00000028982
14	46027689	G	A	intron_variant	MODIFIER	c.342+7416C>T	ZNF704	protein_coding	ENSBTAT00000028982
14	46027881	T	C	intron_variant	MODIFIER	c.342+7224A>G	ZNF704	protein_coding	ENSBTAT00000028982
14	46028081	G	A	intron_variant	MODIFIER	c.342+7024C>T	ZNF704	protein_coding	ENSBTAT00000028982
14	46028229	C	T	intron_variant	MODIFIER	c.342+6876G>A	ZNF704	protein_coding	ENSBTAT00000028982
14	46028353	T	G	intron_variant	MODIFIER	c.342+6752A>C	ZNF704	protein_coding	ENSBTAT00000028982
14	46028450	A	T	intron_variant	MODIFIER	c.342+6655T>A	ZNF704	protein_coding	ENSBTAT00000028982
14	46028545	T	G	intron_variant	MODIFIER	c.342+6560A>C	ZNF704	protein_coding	ENSBTAT00000028982
14	46029030	C	T	intron_variant	MODIFIER	c.342+6075G>A	ZNF704	protein_coding	ENSBTAT00000028982
14	46029033	C	A	intron_variant	MODIFIER	c.342+6072G>T	ZNF704	protein_coding	ENSBTAT00000028982
14	46029790	C	G	intron_variant	MODIFIER	c.342+5315G>C	ZNF704	protein_coding	ENSBTAT00000028982
14	46029914	G	C	intron_variant	MODIFIER	c.342+5191C>G	ZNF704	protein_coding	ENSBTAT00000028982
14	46029957	G	T	intron_variant	MODIFIER	c.342+5148C>A	ZNF704	protein_coding	ENSBTAT00000028982
14	46030077	G	T	intron_variant	MODIFIER	c.342+5028C>A	ZNF704	protein_coding	ENSBTAT00000028982
14	46030206	T	A	intron_variant	MODIFIER	c.342+4899A>T	ZNF704	protein_coding	ENSBTAT00000028982
14	46030207	C	A	intron_variant	MODIFIER	c.342+4898G>T	ZNF704	protein_coding	ENSBTAT00000028982
14	46030277	T	C	intron_variant	MODIFIER	c.342+4828A>G	ZNF704	protein_coding	ENSBTAT00000028982
14	46030480	C	A	intron_variant	MODIFIER	c.342+4625G>T	ZNF704	protein_coding	ENSBTAT00000028982
14	46030831	T	C	intron_variant	MODIFIER	c.342+4274A>G	ZNF704	protein_coding	ENSBTAT00000028982
14	46031123	A	G	intron_variant	MODIFIER	c.342+3982T>C	ZNF704	protein_coding	ENSBTAT00000028982
14	46031476	T	C	intron_variant	MODIFIER	c.342+3629A>G	ZNF704	protein_coding	ENSBTAT00000028982
14	46031528	C	T	intron_variant	MODIFIER	c.342+3577G>A	ZNF704	protein_coding	ENSBTAT00000028982
14	46032077	C	A	intron_variant	MODIFIER	c.342+3028G>T	ZNF704	protein_coding	ENSBTAT00000028982
14	46032078	C	T	intron_variant	MODIFIER	c.342+3027G>A	ZNF704	protein_coding	ENSBTAT00000028982
14	46032315	G	T	intron_variant	MODIFIER	c.342+2790C>A	ZNF704	protein_coding	ENSBTAT00000028982
14	46032338	C	T	intron_variant	MODIFIER	c.342+2767G>A	ZNF704	protein_coding	ENSBTAT00000028982
14	46032392	T	G	intron_variant	MODIFIER	c.342+2713A>C	ZNF704	protein_coding	ENSBTAT00000028982
14	46032426	T	G	intron_variant	MODIFIER	c.342+2679A>C	ZNF704	protein_coding	ENSBTAT00000028982
14	46032436	T	C	intron_variant	MODIFIER	c.342+2669A>G	ZNF704	protein_coding	ENSBTAT00000028982
14	46032508	T	C	intron_variant	MODIFIER	c.342+2597A>G	ZNF704	protein_coding	ENSBTAT00000028982
14	46032602	A	G	intron_variant	MODIFIER	c.342+2503T>C	ZNF704	protein_coding	ENSBTAT00000028982
14	46032689	G	A	intron_variant	MODIFIER	c.342+2416C>T	ZNF704	protein_coding	ENSBTAT00000028982
14	46032701	C	A	intron_variant	MODIFIER	c.342+2404G>T	ZNF704	protein_coding	ENSBTAT00000028982
14	46032976	G	A	intron_variant	MODIFIER	c.342+2129C>T	ZNF704	protein_coding	ENSBTAT00000028982
14	46033008	G	C	intron_variant	MODIFIER	c.342+2097C>G	ZNF704	protein_coding	ENSBTAT00000028982
14	46033030	C	T	intron_variant	MODIFIER	c.342+2075G>A	ZNF704	protein_coding	ENSBTAT00000028982
14	46033220	C	CA	intron_variant	MODIFIER	c.342+1884_342+1885insT	ZNF704	protein_coding	ENSBTAT00000028982
14	46033563	C	CAG	intron_variant	MODIFIER	c.342+1541_342+1542insCT	ZNF704	protein_coding	ENSBTAT00000028982
14	46033915	G	A	intron_variant	MODIFIER	c.342+1190C>T	ZNF704	protein_coding	ENSBTAT00000028982
14	46034026	T	C	intron_variant	MODIFIER	c.342+1079A>G	ZNF704	protein_coding	ENSBTAT00000028982
14	46034233	T	G	intron_variant	MODIFIER	c.342+872A>C	ZNF704	protein_coding	ENSBTAT00000028982
14	46034690	T	G	intron_variant	MODIFIER	c.342+415A>C	ZNF704	protein_coding	ENSBTAT00000028982
14	46034730	GT	G	intron_variant	MODIFIER	c.342+374delA	ZNF704	protein_coding	ENSBTAT00000028982
14	46034825	T	G	intron_variant	MODIFIER	c.342+280A>C	ZNF704	protein_coding	ENSBTAT00000028982
14	46034917	G	C	intron_variant	MODIFIER	c.342+188C>G	ZNF704	protein_coding	ENSBTAT00000028982
14	46034921	T	G	intron_variant	MODIFIER	c.342+184A>C	ZNF704	protein_coding	ENSBTAT00000028982
14	46035093	T	C	intron_variant	MODIFIER	c.342+12A>G	ZNF704	protein_coding	ENSBTAT00000028982
14	46036188	T	C	intron_variant	MODIFIER	c.110-851A>G	ZNF704	protein_coding	ENSBTAT00000028982
14	46036420	A	G	intron_variant	MODIFIER	c.110-1083T>C	ZNF704	protein_coding	ENSBTAT00000028982
14	46036443	T	C	intron_variant	MODIFIER	c.110-1106A>G	ZNF704	protein_coding	ENSBTAT00000028982
14	46036684	C	T	intron_variant	MODIFIER	c.110-1347G>A	ZNF704	protein_coding	ENSBTAT00000028982
14	46036848	A	AACAC	intron_variant	MODIFIER	c.110-1512_110-1511insGTGT	ZNF704	protein_coding	ENSBTAT00000028982
14	46036850	G	A	intron_variant	MODIFIER	c.110-1513C>T	ZNF704	protein_coding	ENSBTAT00000028982
14	46036892	C	T	intron_variant	MODIFIER	c.110-1555G>A	ZNF704	protein_coding	ENSBTAT00000028982
14	46036912	A	G	intron_variant	MODIFIER	c.110-1575T>C	ZNF704	protein_coding	ENSBTAT00000028982
14	46036938	G	A	intron_variant	MODIFIER	c.110-1601C>T	ZNF704	protein_coding	ENSBTAT00000028982
14	46037179	C	T	intron_variant	MODIFIER	c.110-1842G>A	ZNF704	protein_coding	ENSBTAT00000028982
14	46037187	A	G	intron_variant	MODIFIER	c.110-1850T>C	ZNF704	protein_coding	ENSBTAT00000028982
14	46037233	C	T	intron_variant	MODIFIER	c.110-1896G>A	ZNF704	protein_coding	ENSBTAT00000028982
14	46037553	T	C	intron_variant	MODIFIER	c.110-2216A>G	ZNF704	protein_coding	ENSBTAT00000028982
14	46037599	C	T	intron_variant	MODIFIER	c.110-2262G>A	ZNF704	protein_coding	ENSBTAT00000028982
14	46037603	C	A	intron_variant	MODIFIER	c.110-2266G>T	ZNF704	protein_coding	ENSBTAT00000028982
14	46037666	T	C	intron_variant	MODIFIER	c.110-2329A>G	ZNF704	protein_coding	ENSBTAT00000028982
14	46037836	T	G	intron_variant	MODIFIER	c.110-2499A>C	ZNF704	protein_coding	ENSBTAT00000028982
14	46037891	C	T	intron_variant	MODIFIER	c.110-2554G>A	ZNF704	protein_coding	ENSBTAT00000028982
14	46038002	A	C	intron_variant	MODIFIER	c.109+2650T>G	ZNF704	protein_coding	ENSBTAT00000028982
14	46038304	T	C	intron_variant	MODIFIER	c.109+2348A>G	ZNF704	protein_coding	ENSBTAT00000028982
14	46038319	T	C	intron_variant	MODIFIER	c.109+2333A>G	ZNF704	protein_coding	ENSBTAT00000028982
14	46038622	G	T	intron_variant	MODIFIER	c.109+2030C>A	ZNF704	protein_coding	ENSBTAT00000028982
14	46038814	A	T	intron_variant	MODIFIER	c.109+1838T>A	ZNF704	protein_coding	ENSBTAT00000028982
14	46038820	T	C	intron_variant	MODIFIER	c.109+1832A>G	ZNF704	protein_coding	ENSBTAT00000028982
14	46038821	G	A	intron_variant	MODIFIER	c.109+1831C>T	ZNF704	protein_coding	ENSBTAT00000028982
14	46039034	T	C	intron_variant	MODIFIER	c.109+1618A>G	ZNF704	protein_coding	ENSBTAT00000028982
14	46039260	A	G	intron_variant	MODIFIER	c.109+1392T>C	ZNF704	protein_coding	ENSBTAT00000028982
14	46039406	G	A	intron_variant	MODIFIER	c.109+1246C>T	ZNF704	protein_coding	ENSBTAT00000028982
14	46039413	T	C	intron_variant	MODIFIER	c.109+1239A>G	ZNF704	protein_coding	ENSBTAT00000028982
14	46039488	G	A	intron_variant	MODIFIER	c.109+1164C>T	ZNF704	protein_coding	ENSBTAT00000028982
14	46039555	TC	T	intron_variant	MODIFIER	c.109+1096delG	ZNF704	protein_coding	ENSBTAT00000028982
14	46039603	A	G	intron_variant	MODIFIER	c.109+1049T>C	ZNF704	protein_coding	ENSBTAT00000028982
14	46039656	C	T	intron_variant	MODIFIER	c.109+996G>A	ZNF704	protein_coding	ENSBTAT00000028982
14	46039694	TC	T	intron_variant	MODIFIER	c.109+957delG	ZNF704	protein_coding	ENSBTAT00000028982
14	46039745	C	T	intron_variant	MODIFIER	c.109+907G>A	ZNF704	protein_coding	ENSBTAT00000028982
14	46039838	C	T	intron_variant	MODIFIER	c.109+814G>A	ZNF704	protein_coding	ENSBTAT00000028982
14	46040027	A	G	intron_variant	MODIFIER	c.109+625T>C	ZNF704	protein_coding	ENSBTAT00000028982
14	46040421	A	C	intron_variant	MODIFIER	c.109+231T>G	ZNF704	protein_coding	ENSBTAT00000028982
14	46041317	A	T	upstream_gene_variant	MODIFIER	c.-557T>A	ZNF704	protein_coding	ENSBTAT00000028982
14	46041737	T	G	upstream_gene_variant	MODIFIER	c.-977A>C	ZNF704	protein_coding	ENSBTAT00000028982
14	46042459	G	A	upstream_gene_variant	MODIFIER	c.-1699C>T	ZNF704	protein_coding	ENSBTAT00000028982
14	46043072	G	C	upstream_gene_variant	MODIFIER	c.-2312C>G	ZNF704	protein_coding	ENSBTAT00000028982
14	46043383	A	G	upstream_gene_variant	MODIFIER	c.-2623T>C	ZNF704	protein_coding	ENSBTAT00000028982
14	46043446	T	G	upstream_gene_variant	MODIFIER	c.-2686A>C	ZNF704	protein_coding	ENSBTAT00000028982
14	46043588	T	C	upstream_gene_variant	MODIFIER	c.-2828A>G	ZNF704	protein_coding	ENSBTAT00000028982
14	46043886	T	C	upstream_gene_variant	MODIFIER	c.-3126A>G	ZNF704	protein_coding	ENSBTAT00000028982
14	46043899	T	C	upstream_gene_variant	MODIFIER	c.-3139A>G	ZNF704	protein_coding	ENSBTAT00000028982
14	46044196	T	C	upstream_gene_variant	MODIFIER	c.-3436A>G	ZNF704	protein_coding	ENSBTAT00000028982
14	46044335	A	G	upstream_gene_variant	MODIFIER	c.-3575T>C	ZNF704	protein_coding	ENSBTAT00000028982
14	46044439	C	T	upstream_gene_variant	MODIFIER	c.-3679G>A	ZNF704	protein_coding	ENSBTAT00000028982
14	46044459	C	T	upstream_gene_variant	MODIFIER	c.-3699G>A	ZNF704	protein_coding	ENSBTAT00000028982
14	46044793	G	C	upstream_gene_variant	MODIFIER	c.-4033C>G	ZNF704	protein_coding	ENSBTAT00000028982
14	46044922	A	G	upstream_gene_variant	MODIFIER	c.-4162T>C	ZNF704	protein_coding	ENSBTAT00000028982
14	46045386	A	G	upstream_gene_variant	MODIFIER	c.-4626T>C	ZNF704	protein_coding	ENSBTAT00000028982
14	46045522	G	A	upstream_gene_variant	MODIFIER	c.-4762C>T	ZNF704	protein_coding	ENSBTAT00000028982
14	46045553	G	C	upstream_gene_variant	MODIFIER	c.-4793C>G	ZNF704	protein_coding	ENSBTAT00000028982
14	46045629	A	AC	upstream_gene_variant	MODIFIER	c.-4870_-4869insG	ZNF704	protein_coding	ENSBTAT00000028982
14	46799575	G	A	downstream_gene_variant	MODIFIER	c.*3229C>T	PMP2	protein_coding	ENSBTAT00000046521
17	28634949	T	C	upstream_gene_variant	MODIFIER	n.-2214A>G	7SK	misc_RNA	ENSBTAT00000060712

17	28634973	A	G	upstream_gene_variant	MODIFIER	n.-2238T>C	7SK	misc_RNA	ENSBTAT00000060712
17	38028857	T	G	downstream_gene_variant	MODIFIER	c.*67146T>G	ENSBTAG00000047595	protein_coding	ENSBTAT00000064567
17	52969236	G	A	downstream_gene_variant	MODIFIER	c.*5947C>T	AACS	protein_coding	ENSBTAT00000007459
17	52970236	C	T	downstream_gene_variant	MODIFIER	c.*4947G>A	AACS	protein_coding	ENSBTAT00000007459
17	53003481	G	A	intron_variant	MODIFIER	c.571-1375C>T	AACS	protein_coding	ENSBTAT00000007459
17	53009053	G	A	intron_variant	MODIFIER	c.570+2151C>T	AACS	protein_coding	ENSBTAT00000007459
17	53640197	C	T	intron_variant	MODIFIER	c.1286+4889C>T	NCOR2	protein_coding	ENSBTAT00000034273
17	54379369	C	T	intron_variant	MODIFIER	c.801+3770C>T	RILPL1	protein_coding	ENSBTAT00000029668
17	54999680	C	T	upstream_gene_variant	MODIFIER	n.-2052G>A	SNORA70	snoRNA	ENSBTAT00000062459
17	54999680	C	T	intron_variant	MODIFIER	c.212-259G>A	ENSBTAG00000032433	protein_coding	ENSBTAT00000045998
17	55636176	T	C	intron_variant	MODIFIER	c.1581-632A>G	WDR66	protein_coding	ENSBTAT00000005477
17	56675700	G	A	intron_variant	MODIFIER	c.233-4781C>T	PPTC7	protein_coding	ENSBTAT00000024393
17	57292132	T	A	intron_variant	MODIFIER	c.301+9483T>A	CUX2	protein_coding	ENSBTAT00000029615
17	57977763	G	A	intron_variant	MODIFIER	c.5209-82G>A	CIT	protein_coding	ENSBTAT00000061505
17	59716009	C	T	intron_variant	MODIFIER	c.987-20520C>T	KSR2	protein_coding	ENSBTAT00000061442
17	60092232	AC	A	intron_variant	MODIFIER	c.1664+1060delC	NOS1	protein_coding	ENSBTAT00000011690
17	60335279	C	T	downstream_gene_variant	MODIFIER	c.*3729C>T	TESC	protein_coding	ENSBTAT00000061490
17	60335279	C	T	downstream_gene_variant	MODIFIER	c.*3858G>A	FBXW8	protein_coding	ENSBTAT00000064959
17	60335279	C	T	downstream_gene_variant	MODIFIER	c.*3858G>A	FBXW8	protein_coding	ENSBTAT00000050675
18	14609330	C	T	upstream_gene_variant	MODIFIER	c.-1437C>T	CDK10	protein_coding	ENSBTAT00000047400
18	14611199	C	T	intron_variant	MODIFIER	c.81+352C>T	CDK10	protein_coding	ENSBTAT00000047400
18	14628123	TGGGGTCACA	T	intron_variant	MODIFIER	c.1282+43_1282+51delTGTGACCCC	VPS9D1	protein_coding	ENSBTAT00000028205
18	14632547	C	T	upstream_gene_variant	MODIFIER	c.-2755C>T	ZNF276	protein_coding	ENSBTAT00000002476
18	14632547	C	T	intron_variant	MODIFIER	c.175+329G>A	VPS9D1	protein_coding	ENSBTAT00000028205
18	14639106	A	G	upstream_gene_variant	MODIFIER	c.-4534T>C	VPS9D1	protein_coding	ENSBTAT00000028205
18	14639106	A	G	intron_variant	MODIFIER	c.218-699A>G	ZNF276	protein_coding	ENSBTAT00000002476
18	14669751	C	A	intron_variant	MODIFIER	c.1718+242G>T	FANCA	protein_coding	ENSBTAT00000002478
18	14673330	C	T	intron_variant	MODIFIER	c.1362+301G>A	FANCA	protein_coding	ENSBTAT00000002478
18	14684754	G	A	intron_variant	MODIFIER	c.284-398C>T	FANCA	protein_coding	ENSBTAT00000002478
18	14692800	G	C	upstream_gene_variant	MODIFIER	c.-1652G>C	SPIRE2	protein_coding	ENSBTAT00000001541
18	14692800	G	C	upstream_gene_variant	MODIFIER	c.-1682G>C	SPIRE2	protein_coding	ENSBTAT00000064626
18	14740292	T	TG	upstream_gene_variant	MODIFIER	c.-1645_-1644insG	TCF25	protein_coding	ENSBTAT00000016057
18	14743791	A	G	synonymous_variant	LOW	c.369A>G	TCF25	protein_coding	ENSBTAT00000016057
18	14751873	G	A	intron_variant	MODIFIER	c.1278-142G>A	TCF25	protein_coding	ENSBTAT00000016057
18	14756082	C	T	upstream_gene_variant	MODIFIER	c.-1533C>T	MC1R	protein_coding	ENSBTAT00000032494
18	14756082	C	T	downstream_gene_variant	MODIFIER	c.*2181C>T	TCF25	protein_coding	ENSBTAT00000016057
18	14796948	G	A	intron_variant	MODIFIER	c.139-1948G>A	ENSBTAG00000038051	protein_coding	ENSBTAT00000056979
18	14802791	A	G	intron_variant	MODIFIER	c.546+655A>G	ENSBTAG00000038051	protein_coding	ENSBTAT00000056979
18	14802810	T	C	intron_variant	MODIFIER	c.546+674T>C	ENSBTAG00000038051	protein_coding	ENSBTAT00000056979
18	14803413	G	C	intron_variant	MODIFIER	c.547-861G>C	ENSBTAG00000038051	protein_coding	ENSBTAT00000056979
18	14806770	G	A	intron_variant	MODIFIER	c.1011+1041G>A	ENSBTAG00000038051	protein_coding	ENSBTAT00000056979
18	14820167	C	T	downstream_gene_variant	MODIFIER	c.*4957C>T	ENSBTAG00000038051	protein_coding	ENSBTAT00000056979
18	14820167	C	T	intron_variant	MODIFIER	c.32-892G>A	DBNDD1	protein_coding	ENSBTAT00000027077
18	14822098	G	A	intron_variant	MODIFIER	c.31+2562C>T	DBNDD1	protein_coding	ENSBTAT00000027077
18	14830472	C	CT	intron_variant	MODIFIER	c.3+127_3+128insT	GAS8	protein_coding	ENSBTAT00000009333
18	14846463	C	T	3_prime_UTR_variant	MODIFIER	c.*263C>T	GAS8	protein_coding	ENSBTAT00000009333
18	14846786	C	T	3_prime_UTR_variant	MODIFIER	c.*586C>T	GAS8	protein_coding	ENSBTAT00000009333
18	14849211	G	A	downstream_gene_variant	MODIFIER	c.*3371G>A	GAS8	protein_coding	ENSBTAT00000009333
18	14849326	C	T	downstream_gene_variant	MODIFIER	c.*3486C>T	GAS8	protein_coding	ENSBTAT00000009333
18	14882790	C	T	upstream_gene_variant	MODIFIER	n.-4945G>A	U1	snRNA	ENSBTAT00000042019
18	15077671	T	A	upstream_gene_variant	MODIFIER	c.-822T>A	ENSBTAG00000023726	protein_coding	ENSBTAT00000032490
18	15077671	T	A	downstream_gene_variant	MODIFIER	c.*2815T>A	ORC6	protein_coding	ENSBTAT00000002438
18	15406139	C	A	downstream_gene_variant	MODIFIER	c.*1537C>A	GPT2	protein_coding	ENSBTAT00000003881
19	17685195	A	G	intron_variant	MODIFIER	c.95+19650A>G	MYO1D	protein_coding	ENSBTAT00000020634
19	17793549	AT	A	intron_variant	MODIFIER	c.1297-552delT	MYO1D	protein_coding	ENSBTAT00000020634
19	18272256	G	T	intron_variant	MODIFIER	c.1139+538C>A	RHOT1	protein_coding	ENSBTAT00000035318
19	18292007	C	G	intron_variant	MODIFIER	c.368+578G>C	RHOT1	protein_coding	ENSBTAT00000035318
19	18397920	G	A	upstream_gene_variant	MODIFIER	c.-2837G>A	C19H17orf42	protein_coding	ENSBTAT00000024467
19	61710902	AAAAAG	A	intron_variant	MODIFIER	c.928-1804_928-1800delCTTTT	MAP2K6	protein_coding	ENSBTAT00000002115
19	61710907	G	A	intron_variant	MODIFIER	c.928-1804C>T	MAP2K6	protein_coding	ENSBTAT00000002115
19	61748890	A	G	intron_variant	MODIFIER	c.17-5190T>C	MAP2K6	protein_coding	ENSBTAT00000002115
19	61777308	G	A	intron_variant	MODIFIER	c.17-33608C>T	MAP2K6	protein_coding	ENSBTAT00000002115
19	62274726	C	T	intron_variant	MODIFIER	c.356+4098C>T	FAM20A	protein_coding	ENSBTAT00000001439
21	55537362	T	G	intron_variant	MODIFIER	c.862+1531A>C	TGM5	protein_coding	ENSBTAT00000045466
21	55625460	C	T	splice_region_variant&intron_variant	LOW	c.770-8C>T	ADAL	protein_coding	ENSBTAT00000019248
21	55625460	C	T	downstream_gene_variant	MODIFIER	c.*4898G>A	ZSCAN29	protein_coding	ENSBTAT00000019251
21	55707719	T	C	intron_variant	MODIFIER	c.3627+608A>G	TP53BP1	protein_coding	ENSBTAT00000028388
21	55708316	T	C	intron_variant	MODIFIER	c.3627+11A>G	TP53BP1	protein_coding	ENSBTAT00000028388
23	11960224	TAA	T	intron_variant	MODIFIER	c.113-1005_113-1004delAA	ZFAND3	protein_coding	ENSBTAT00000036100
23	12480092	C	A	downstream_gene_variant	MODIFIER	c.*4750G>T	GLO1	protein_coding	ENSBTAT00000016884
23	12620146	G	A	intron_variant	MODIFIER	c.2965-4714G>A	DNAH8	protein_coding	ENSBTAT00000018689
23	12620146	G	A	intron_variant	MODIFIER	c.2569-4714G>A	DNAH8	protein_coding	ENSBTAT00000065917
23	12622781	C	A	intron_variant	MODIFIER	c.2965-2079C>A	DNAH8	protein_coding	ENSBTAT00000018689
23	12622781	C	A	intron_variant	MODIFIER	c.2569-2079C>A	DNAH8	protein_coding	ENSBTAT00000065917
23	12711687	C	G	intron_variant	MODIFIER	c.8331+494C>G	DNAH8	protein_coding	ENSBTAT00000018689
23	12711687	C	G	intron_variant	MODIFIER	c.7983+494C>G	DNAH8	protein_coding	ENSBTAT00000065917
23	13071905	G	A	intron_variant	MODIFIER	c.187-9651C>T	KCNK5	protein_coding	ENSBTAT00000014756
23	13104027	C	T	upstream_gene_variant	MODIFIER	c.-801G>A	ENSBTAG00000007140	protein_coding	ENSBTAT00000009396
23	13481679	G	T	intron_variant	MODIFIER	c.391-1303C>A	KIF6	protein_coding	ENSBTAT00000064002
23	13481679	G	T	intron_variant	MODIFIER	c.391-1303C>A	KIF6	protein_coding	ENSBTAT00000009903
23	13481679	G	T	intron_variant	MODIFIER	c.391-1303C>A	KIF6	protein_coding	ENSBTAT00000064399
23	13793289	A	G	intron_variant	MODIFIER	c.796-394A>G	DAAM2	protein_coding	ENSBTAT00000036988
24	3880273	T	C	intron_variant	MODIFIER	c.5333-37941A>G	ZNF407	protein_coding	ENSBTAT00000031106
24	4093660	G	A	intron_variant	MODIFIER	c.4701-17426C>T	ZNF407	protein_coding	ENSBTAT00000031106
24	4150517	CATT	C	intron_variant	MODIFIER	c.4700+37021_4700+37023delAAT	ZNF407	protein_coding	ENSBTAT00000031106
24	4261031	G	A	upstream_gene_variant	MODIFIER	c.-5918C>T	CNDP1	protein_coding	ENSBTAT00000028564
24	4553586	T	G	intron_variant	MODIFIER	c.1007-4409T>G	FBXO15	protein_coding	ENSBTAT00000012776
24	21008218	A	G	intron_variant	MODIFIER	c.338-66972T>C	FHOD3	protein_coding	ENSBTAT00000061003
24	21153591	G	A	downstream_gene_variant	MODIFIER	n.*1089C>T	U6	snRNA	ENSBTAT00000059186
24	21153591	G	A	intron_variant	MODIFIER	c.165+18271C>T	FHOD3	protein_coding	ENSBTAT00000061003
24	21171558	G	C	intron_variant	MODIFIER	c.165+304C>G	FHOD3	protein_coding	ENSBTAT00000061003
**24**	**21222029**	**TC**	**T**	**frameshift_variant**	**HIGH**	**c.1881delG**	**MOCOS**	**protein_coding**	**ENSBTAT00000065375**
**24**	**21222029**	**TC**	**T**	**frameshift_variant**	**HIGH**	**c.1782delG**	**MOCOS**	**protein_coding**	**ENSBTAT00000048768**
24	21923967	T	A	downstream_gene_variant	MODIFIER	c.*2734T>A	INO80C	protein_coding	ENSBTAT00000065749
24	21923967	T	A	downstream_gene_variant	MODIFIER	c.*2734T>A	INO80C	protein_coding	ENSBTAT00000016489
24	22077117	A	G	upstream_gene_variant	MODIFIER	c.-3015A>G	ZNF24	protein_coding	ENSBTAT00000013547
24	22077117	A	G	intron_variant	MODIFIER	c.1261-10835A>G	ZNF24	protein_coding	ENSBTAT00000065887
24	22077117	A	G	intron_variant	MODIFIER	c.794+17811T>C	ZNF397	protein_coding	ENSBTAT00000065936
24	22099857	CA	C	upstream_gene_variant	MODIFIER	c.-4137delT	ZNF397	protein_coding	ENSBTAT00000065936
24	22099857	CA	C	intron_variant	MODIFIER	c.-81-487delT	ZNF397	protein_coding	ENSBTAT00000013542
24	22235790	G	A	intron_variant	MODIFIER	c.394-1265C>T	MAPRE2	protein_coding	ENSBTAT00000009897
24	22279375	TC	T	intron_variant	MODIFIER	c.120-6449delG	MAPRE2	protein_coding	ENSBTAT00000009897
24	22338374	C	T	upstream_gene_variant	MODIFIER	n.-1591G>A	5S_rRNA	rRNA	ENSBTAT00000060885
24	24745064	T	C	intron_variant	MODIFIER	c.1064-20728T>C	KLHL14	protein_coding	ENSBTAT00000026649
24	24745227	T	A	intron_variant	MODIFIER	c.1064-20565T>A	KLHL14	protein_coding	ENSBTAT00000026649
24	24747332	G	A	intron_variant	MODIFIER	c.1064-18460G>A	KLHL14	protein_coding	ENSBTAT00000026649
24	29069651	A	G	intron_variant	MODIFIER	c.172+45245A>G	CDH2	protein_coding	ENSBTAT00000028238
24	29069652	A	G	intron_variant	MODIFIER	c.172+45246A>G	CDH2	protein_coding	ENSBTAT00000028238
24	29069653	A	G	intron_variant	MODIFIER	c.172+45247A>G	CDH2	protein_coding	ENSBTAT00000028238
24	29069654	A	G	intron_variant	MODIFIER	c.172+45248A>G	CDH2	protein_coding	ENSBTAT00000028238
24	30224398	T	C	intron_variant	MODIFIER	c.163+20818T>C	CHST9	protein_coding	ENSBTAT00000031067
24	30231236	T	C	intron_variant	MODIFIER	c.163+27656T>C	CHST9	protein_coding	ENSBTAT00000031067
24	31055243	A	G	downstream_gene_variant	MODIFIER	n.*363A>G	bta-mir-2380	miRNA	ENSBTAT00000062477

24	31055243	A	G	intron_variant	MODIFIER	c.232-3475A>G	SS18	protein_coding	ENSBTAT00000015327
24	31058279	C	A	downstream_gene_variant	MODIFIER	n.*3399C>A	bta-mir-2380	miRNA	ENSBTAT00000062477
24	31058279	C	A	intron_variant	MODIFIER	c.232-439C>A	SS18	protein_coding	ENSBTAT00000015327
24	31778728	G	T	intron_variant	MODIFIER	c.220+72614G>T	ZNF521	protein_coding	ENSBTAT00000048222
24	31960710	A	C	intron_variant	MODIFIER	c.3658+55508A>C	ZNF521	protein_coding	ENSBTAT00000048222
24	57302555	C	T	intron_variant	MODIFIER	c.784-203G>A	FECH	protein_coding	ENSBTAT00000008384
24	58149589	C	T	intron_variant	MODIFIER	c.5536+767G>A	ALPK2	protein_coding	ENSBTAT00000018977
24	58171663	G	A	intron_variant	MODIFIER	c.1885-10367C>T	ALPK2	protein_coding	ENSBTAT00000018977
24	58292677	C	T	intron_variant	MODIFIER	c.228-4364C>T	MALT1	protein_coding	ENSBTAT00000046385
27	33752338	G	A	intron_variant	MODIFIER	c.61-2392G>A	PLEKHA2	protein_coding	ENSBTAT00000031486
27	34689026	C	T	intron_variant	MODIFIER	c.100-2006C>T	IDO1	protein_coding	ENSBTAT00000027449
27	34695686	T	C	intron_variant	MODIFIER	c.550-13T>C	IDO1	protein_coding	ENSBTAT00000027449
27	35335361	C	T	intron_variant	MODIFIER	c.578-36307G>A	ZMAT4	protein_coding	ENSBTAT00000047349
27	35338339	G	A	intron_variant	MODIFIER	c.578-39285C>T	ZMAT4	protein_coding	ENSBTAT00000047349
27	35358557	C	T	intron_variant	MODIFIER	c.577+22579G>A	ZMAT4	protein_coding	ENSBTAT00000047349
27	35365674	G	A	intron_variant	MODIFIER	c.577+15462C>T	ZMAT4	protein_coding	ENSBTAT00000047349
27	35418117	C	T	intron_variant	MODIFIER	c.193-8821G>A	ZMAT4	protein_coding	ENSBTAT00000047349
27	35418155	C	A	intron_variant	MODIFIER	c.193-8859G>T	ZMAT4	protein_coding	ENSBTAT00000047349
