# Supplementary material for: A frameshift mutation in MOCOS is associated with familial renal syndrome (xanthinuria) in Tyrolean Grey cattle
Source: BMC Vet Res. 2016 Dec 5;12:276. doi: 10.1186/s12917-016-0904-4 (PMC5139135; doi:10.1186/s12917-016-0904-4)
Supplement: Additional file 3: — List of private heterozygous variants in annotated genes found in the genome of the sequenced animal. (PDF 31 kb) [file 12917_2016_904_MOESM3_ESM.pdf]

Additional file 3:

List of private heterozygous variants in annotated genes found in the genome of the sequenced animal.

Chromosome	*Position*	*REF*	*ALT*	*EFFECT*	*IMPACT*	*CODON*	*GENE*	*BIOTYPE*	*TRID*
2	44565696	T	C	synonymous_variant	LOW	c.882T>C	NEB	protein_coding	ENSBTAT00000061000
2	44565696	T	C	synonymous_variant	LOW	c.882T>C	NEB	protein_coding	ENSBTAT00000055345
2	44593113	C	G	synonymous_variant	LOW	c.3462C>G	NEB	protein_coding	ENSBTAT00000061000
2	44593113	C	G	synonymous_variant	LOW	c.3462C>G	NEB	protein_coding	ENSBTAT00000055345
2	44620609	A	G	synonymous_variant	LOW	c.7206A>G	NEB	protein_coding	ENSBTAT00000061000
2	44620609	A	G	synonymous_variant	LOW	c.7206A>G	NEB	protein_coding	ENSBTAT00000055345
2	44620947	A	G	synonymous_variant	LOW	c.7338A>G	NEB	protein_coding	ENSBTAT00000061000
2	44620947	A	G	synonymous_variant	LOW	c.7338A>G	NEB	protein_coding	ENSBTAT00000055345
2	44621016	T	C	synonymous_variant	LOW	c.7407T>C	NEB	protein_coding	ENSBTAT00000061000
2	44621016	T	C	synonymous_variant	LOW	c.7407T>C	NEB	protein_coding	ENSBTAT00000055345
2	44621033	C	T	splice_region_variant&intron_variant	LOW	c.7416+8C>T	NEB	protein_coding	ENSBTAT00000061000
2	44621033	C	T	splice_region_variant&intron_variant	LOW	c.7416+8C>T	NEB	protein_coding	ENSBTAT00000055345
2	44624707	T	C	synonymous_variant	LOW	c.7494T>C	NEB	protein_coding	ENSBTAT00000061000
2	44624707	T	C	synonymous_variant	LOW	c.7494T>C	NEB	protein_coding	ENSBTAT00000055345
2	44627138	T	C	synonymous_variant	LOW	c.7956T>C	NEB	protein_coding	ENSBTAT00000061000
2	44627138	T	C	synonymous_variant	LOW	c.7956T>C	NEB	protein_coding	ENSBTAT00000055345
2	44631045	G	A	synonymous_variant	LOW	c.8958G>A	NEB	protein_coding	ENSBTAT00000061000
2	44631045	G	A	intron_variant	MODIFIER	c.8874+544G>A	NEB	protein_coding	ENSBTAT00000055345
2	44634898	C	T	synonymous_variant	LOW	c.9075C>T	NEB	protein_coding	ENSBTAT00000061000
2	44634898	C	T	intron_variant	MODIFIER	c.8875-4149C>T	NEB	protein_coding	ENSBTAT00000055345
2	44635982	C	T	synonymous_variant	LOW	c.9180C>T	NEB	protein_coding	ENSBTAT00000061000
2	44635982	C	T	intron_variant	MODIFIER	c.8875-3065C>T	NEB	protein_coding	ENSBTAT00000055345
2	44640539	A	G	synonymous_variant	LOW	c.9714A>G	NEB	protein_coding	ENSBTAT00000061000
2	44640539	A	G	synonymous_variant	LOW	c.8985A>G	NEB	protein_coding	ENSBTAT00000055345
2	44640585	A	C	synonymous_variant	LOW	c.9760A>C	NEB	protein_coding	ENSBTAT00000061000
2	44640585	A	C	synonymous_variant	LOW	c.9031A>C	NEB	protein_coding	ENSBTAT00000055345
2	44640649	G	C	splice_region_variant&intron_variant	LOW	c.9816+8G>C	NEB	protein_coding	ENSBTAT00000061000
2	44640649	G	C	splice_region_variant&intron_variant	LOW	c.9087+8G>C	NEB	protein_coding	ENSBTAT00000055345
2	44647173	A	G	synonymous_variant	LOW	c.10539A>G	NEB	protein_coding	ENSBTAT00000061000
2	44647173	A	G	synonymous_variant	LOW	c.9810A>G	NEB	protein_coding	ENSBTAT00000055345
2	44648435	C	T	synonymous_variant	LOW	c.10701C>T	NEB	protein_coding	ENSBTAT00000061000
2	44648435	C	T	synonymous_variant	LOW	c.9972C>T	NEB	protein_coding	ENSBTAT00000055345
2	44706664	G	A	splice_region_variant&intron_variant	LOW	c.16510-7G>A	NEB	protein_coding	ENSBTAT00000061000
2	44768187	A	C	synonymous_variant	LOW	c.6831T>G	RIF1	protein_coding	ENSBTAT00000027997
3	86392279	C	G	upstream_gene_variant	MODIFIER	c.-1C>G	ENSBTAG00000038085	protein_coding	ENSBTAT00000051914
3	86392279	C	G	intergenic_region	MODIFIER	n.86392279C>G	C1orf87-ENSBTAG00000038085		
5	25778440	C	A	synonymous_variant	LOW	c.183C>A	ITGA5	protein_coding	ENSBTAT00000018261
5	45203368	G	A	5_prime_UTR_variant	MODIFIER	c.-134C>T	MDM2	protein_coding	ENSBTAT00000045265
7	2593118	C	A	5_prime_UTR_variant	MODIFIER	c.-1967G>T	ENSBTAG00000001604	protein_coding	ENSBTAT00000002103
7	15649741	T	C	synonymous_variant	LOW	c.2058T>C	ENSBTAG00000047507	protein_coding	ENSBTAT00000064948
9	50907637	T	A	splice_region_variant&non_coding_exon_variant	LOW	n.102A>T	U6	snRNA	ENSBTAT00000059759
10	12448140	A	G	5_prime_UTR_premature_start_codon_gain_variant	LOW	c.-272T>C	VWA9	protein_coding	ENSBTAT00000020164
10	12448140	A	G	5_prime_UTR_variant	MODIFIER	c.-272T>C	VWA9	protein_coding	ENSBTAT00000020164
11	1774450	G	A	intron_variant	MODIFIER	c.274-1507G>A	MALL	protein_coding	ENSBTAT00000015763
11	2340729	A	G	3_prime_UTR_variant	MODIFIER	c.*778A>G	CIAO1	protein_coding	ENSBTAT00000020797
11	2340729	A	G	downstream_gene_variant	MODIFIER	c.*2525T>C	SNRNP200	protein_coding	ENSBTAT00000001130
11	2340776	G	A	3_prime_UTR_variant	MODIFIER	c.*825G>A	CIAO1	protein_coding	ENSBTAT00000020797
11	2340776	G	A	downstream_gene_variant	MODIFIER	c.*2478C>T	SNRNP200	protein_coding	ENSBTAT00000001130
11	2340779	C	T	3_prime_UTR_variant	MODIFIER	c.*828C>T	CIAO1	protein_coding	ENSBTAT00000020797
11	2340779	C	T	downstream_gene_variant	MODIFIER	c.*2475G>A	SNRNP200	protein_coding	ENSBTAT00000001130
11	2341033	T	C	3_prime_UTR_variant	MODIFIER	c.*1082T>C	CIAO1	protein_coding	ENSBTAT00000020797
11	2341033	T	C	downstream_gene_variant	MODIFIER	c.*2221A>G	SNRNP200	protein_coding	ENSBTAT00000001130
11	2341044	T	A	3_prime_UTR_variant	MODIFIER	c.*1093T>A	CIAO1	protein_coding	ENSBTAT00000020797
11	2341044	T	A	downstream_gene_variant	MODIFIER	c.*2210A>T	SNRNP200	protein_coding	ENSBTAT00000001130
11	2341057	G	A	3_prime_UTR_variant	MODIFIER	c.*1106G>A	CIAO1	protein_coding	ENSBTAT00000020797
11	2341057	G	A	downstream_gene_variant	MODIFIER	c.*2197C>T	SNRNP200	protein_coding	ENSBTAT00000001130
11	2345065	A	C	splice_region_variant&intron_variant	LOW	c.5755-5T>G	SNRNP200	protein_coding	ENSBTAT00000001130
11	2345065	A	C	downstream_gene_variant	MODIFIER	c.*5114A>C	CIAO1	protein_coding	ENSBTAT00000020797
11	2390619	T	C	upstream_gene_variant	MODIFIER	c.-4140T>C	ITPRIPL1	protein_coding	ENSBTAT00000049472
11	2390619	T	C	non_coding_exon_variant	MODIFIER	n.276T>C	ENSBTAG00000047659	processed_pseudogene	ENSBTAT00000065351
11	2390688	A	G	upstream_gene_variant	MODIFIER	c.-4071A>G	ITPRIPL1	protein_coding	ENSBTAT00000049472
11	2390688	A	G	non_coding_exon_variant	MODIFIER	n.345A>G	ENSBTAG00000047659	processed_pseudogene	ENSBTAT00000065351
11	2390800	G	A	upstream_gene_variant	MODIFIER	c.-3959G>A	ITPRIPL1	protein_coding	ENSBTAT00000049472
11	2390800	G	A	downstream_gene_variant	MODIFIER	n.*1G>A	ENSBTAG00000047659	processed_pseudogene	ENSBTAT00000065351
11	2390800	G	A	intergenic_region	MODIFIER	n.2390800G>A	ENSBTAG00000047659-ITPRIPL1		
11	86203078	C	T	synonymous_variant	LOW	c.5337G>A	GREB1	protein_coding	ENSBTAT00000020468
11	99318281	G	A	3_prime_UTR_variant	MODIFIER	c.*20G>A	ENDOG	protein_coding	ENSBTAT00000016564
11	99318281	G	A	downstream_gene_variant	MODIFIER	c.*134C>T	C9orf114	protein_coding	ENSBTAT00000016565
11	103669650	T	C	3_prime_UTR_variant	MODIFIER	c.*328A>G	C9orf69	protein_coding	ENSBTAT00000064693
11	103669915	C	T	3_prime_UTR_variant	MODIFIER	c.*63G>A	C9orf69	protein_coding	ENSBTAT00000064693
11	103758547	G	A	synonymous_variant	LOW	c.555C>T	QSOX2	protein_coding	ENSBTAT00000042859
12	79483263	G	T	splice_region_variant&intron_variant	LOW	c.13-3C>A	STK24	protein_coding	ENSBTAT00000026195
14	18244110	T	G	synonymous_variant	LOW	c.1917A>C	TBC1D31	protein_coding	ENSBTAT00000028197
14	65346798	C	T	3_prime_UTR_variant	MODIFIER	c.*1191C>T	MGC133632	protein_coding	ENSBTAT00000063409
14	65346798	C	T	downstream_gene_variant	MODIFIER	c.*1867C>T	MGC133632	protein_coding	ENSBTAT00000010595
15	48701749	C	T	synonymous_variant	LOW	c.1749C>T	UBQLNL	protein_coding	ENSBTAT00000007451
15	48701749	C	T	upstream_gene_variant	MODIFIER	c.-5270C>T	UBQLN3	protein_coding	ENSBTAT00000035442
16	52336513	T	C	3_prime_UTR_variant	MODIFIER	c.*306A>G	ATAD3	protein_coding	ENSBTAT00000012676
16	52336513	T	C	downstream_gene_variant	MODIFIER	c.*1008T>C	C16H1orf70	protein_coding	ENSBTAT00000065201
16	52336513	T	C	intron_variant	MODIFIER	c.-119+35577A>G	ENSBTAG00000039728	protein_coding	ENSBTAT00000052289
17	45511670	C	T	upstream_gene_variant	MODIFIER	c.-352G>A	PXMP2	protein_coding	ENSBTAT00000009885
17	45511670	C	T	downstream_gene_variant	MODIFIER	n.*1874G>A	U6	snRNA	ENSBTAT00000059869
18	13038986	G	A	synonymous_variant	LOW	c.1299C>T	ZCCHC14	protein_coding	ENSBTAT00000018957
18	14753761	G	T	upstream_gene_variant	MODIFIER	c.-3854G>T	MC1R	protein_coding	ENSBTAT00000032494
19	58500769	C	T	splice_region_variant&intron_variant	LOW	c.1540-3C>T	SDK2	protein_coding	ENSBTAT00000061463
25	7730597	C	T	3_prime_UTR_variant	MODIFIER	c.*1088C>T	PMM2	protein_coding	ENSBTAT00000001888
25	7730597	C	T	downstream_gene_variant	MODIFIER	c.*4239G>A	CARHSP1	protein_coding	ENSBTAT00000001890
27	25556114	C	T	non_coding_exon_variant	MODIFIER	n.268G>A	ENSBTAG00000017570	pseudogene	ENSBTAT00000023357
28	42584826	C	T	non_coding_exon_variant	MODIFIER	n.179C>T	ENSBTAG00000039154	pseudogene	ENSBTAT00000054548
28	42585051	C	T	non_coding_exon_variant	MODIFIER	n.404C>T	ENSBTAG00000039154	pseudogene	ENSBTAT00000054548
28	42585060	G	C	non_coding_exon_variant	MODIFIER	n.413G>C	ENSBTAG00000039154	pseudogene	ENSBTAT00000054548
28	42585151	T	C	non_coding_exon_variant	MODIFIER	n.504T>C	ENSBTAG00000039154	pseudogene	ENSBTAT00000054548
28	42585310	A	G	non_coding_exon_variant	MODIFIER	n.663A>G	ENSBTAG00000039154	pseudogene	ENSBTAT00000054548
29	40703234	G	T	synonymous_variant	LOW	c.867C>A	SYT7	protein_coding	ENSBTAT00000030178
X	80816218	A	G	synonymous_variant	LOW	c.240A>G	ZDHHC15	protein_coding	ENSBTAT00000010589
